# Rapid Evaluation of Chaboche–Voce Material Model Parameters Through a Fully Reversed Strain-Controlled Test

**DOI:** 10.3390/ma19214735

**Published:** 2026-11-07

**Authors:** Alessandro Guazzini, Federico Bucciarelli, Gabriele Tarica, Tommaso Grossi, Bernardo Disma Monelli

**Affiliations:** 1 Department of Civil and Industrial Engineering, University of Pisa, Largo Lucio Lazzarino 1, 56122 Pisa, Italy; gabriele.tarica@phd.unipi.it; 2 Baker Hughes Nuovo Pignone s.r.l, Via Felice Matteucci 2, 50127 Florence, Italy; federico.bucciarelli@bakerhughes.com; 3 TeCIP Institute, Scuola Superiore Sant’Anna, Piazza Martiri della Libertà 33, 56127 Pisa, Italy; tommaso.grossi@santannapisa.it

**Keywords:** cyclic plasticity, high-temperature testing, low-cycle fatigue, Chaboche model, least-squares fitting

## Abstract

Modeling cyclic plasticity remains challenging due to the high computational cost of nonlinear constitutive models and the large number of parameters requiring evaluation, which are often temperature-dependent. This work presents a systematic procedure for identifying the parameters of the Chaboche–Voce model, one of the most widely adopted constitutive models for cyclic plasticity and commonly implemented in finite element software. Due to its nonlinear formulation, however, parameter evaluation is not straightforward. The parameters governing the kinematic hardening response are determined through a simple least-squares fit, yielding an analytical expression for a branch of the mid-life stabilized cycle. The Voce parameters are subsequently obtained by solving an analytical equation followed by a further least-squares fit. The resulting methodology provides a systematic approach for characterizing materials exhibiting both cyclic hardening and softening. The procedure was applied to two materials: 42CrMo4+QT tested at room temperature at different strain ranges for two different values of mean strain and 1Cr-1Mo-0.25V steel tested at different temperatures and strain ranges. The proposed approach provides satisfactory constitutive parameters while requiring virtually negligible computational cost, making it suitable for efficient material characterization and subsequent finite element implementation.

## 1. Introduction

High-temperature and heavy-duty mechanical components made from low-alloy steels are routinely subjected to severe thermo-mechanical cycling during start-up, shutdown, and load-following operation, since they experience a wide range of thermo-mechanical loading during their service life, which can lead to fatigue crack initiation despite static and dynamic design margins. Accurate prediction of Low-Cycle Fatigue (LCF) life in these components requires a constitutive model capable of reproducing the cyclic stress–strain response, including hardening/softening behaviour, mean-stress relaxation, and ratcheting under non-symmetric loading. Since its introduction, the Chaboche nonlinear kinematic hardening model [1,2,3], typically combined with a Voce-type isotropic hardening rule, has become the reference framework thanks to its ability to reproduce the Bauschinger effect, cyclic hardening/softening and ratcheting [4,5,6,7,8,9]. The model represents the superposition of several backstresses, the evolution of which is based on the Armstrong–Frederick law [10].

Despite the wide adoption of the Chaboche–Voce formulation in commercial Finite Element (FE) codes, the practical bottleneck remains the evaluation of its material parameters. Early calibration strategies proposed applying the Ramberg–Osgood equation to the cyclic stress–strain curve to produce a smoothed, extrapolated description of material response, followed by curve fitting. However, when applied to the stabilized cycle, this method is not particularly rigorous, since it ignores the fact that the backstress requires a certain number of cycles to reach its stabilized value. In addition, modeling based on curves obtained using the Ramberg–Osgood model does not provide insight into the cyclic behavior due to the isotropic hardening component [11]. The subsequent literature has moved in two broad directions: on one side, several works have proposed increasingly sophisticated numerical optimization strategies such as gradient-based least-squares fitting, genetic algorithms (GA) and multi-objective GA formulations that simultaneously target hysteresis-loop accuracy and ratcheting prediction [12,13,14,15,16], differential evolution [17] and fuzzy-logic-assisted selection among non-unique parameter sets [18,19,20]. More recently, machine-learning-based surrogates—including artificial neural networks trained to replace costly FE evaluations, and physics-informed neural networks embedding the constitutive equations directly in the loss function—have been used to accelerate or refine the evaluation process [21,22]. On the other side, Bayesian and Markov Chain Monte Carlo (MCMC) approaches have been developed to quantify the uncertainty associated with the estimated parameters, addressing the well-known non-uniqueness of Chaboche parameter sets that fit the same experimental curve almost equally well [23,24].

While these methods have undoubtedly advanced the state of the art, they share two practical limitations that hinder their adoption in an industrial or routine testing context. First, most optimization-based and machine-learning-based procedures require either a large, purpose-built, experimental campaign (multiple strain amplitudes, ratcheting tests, relaxation tests, or full-field measurements), or repeated FE simulations to evaluate the objective function, making them computationally expensive and difficult to reproduce without dedicated software infrastructure. Second, sophisticated black-box procedures such as genetic algorithms and neural-network surrogates typically require careful tuning of hyperparameters (e.g., population size, mutation rate, network architecture). As a consequence, calibration of the Chaboche–Voce model is still often perceived, in practice, as either prohibitively sophisticated for routine material characterization or dependent on ad hoc, material-specific test protocols that are not readily transferable between laboratories. Progress has been made towards developing simplified methodologies that require lower computational effort and smaller experimental campaigns, as reported in Ref. [25]. In that work, analytical relationships describing the half-life cycle and selected physical quantities, such as hysteresis area and asymptotic ratcheting rate, are systematically employed. However, the procedure typically requires the inclusion of cycles with different mean strain values from the outset, owing to potential numerical ill-conditioning that may affect the evaluation of the backstresses. The problem can be overcome through a suggested trial-and-error optimization procedure. Although this limitation can be addressed by designing a targeted experimental campaign, researchers and companies often have to rely on data from previously conducted campaigns, which are typically based on fully reversed strain-controlled tests ($R_{\varepsilon }=-1$) for the determination of the Manson–Coffin curve.

This work proposes a simple and closed-form fitting procedure for the Chaboche–Voce model parameters based only on the theoretical minimum value of the backstress at the stabilized cycle. In addition, it relies on the ascending (or descending) branch of a single stabilized hysteresis loop obtained from only a standard strain-controlled cyclic test. The reason why the proposed methodology does not require additional cycles at different strain ranges for parameter calibration is that it relies on the assumption of Masing behaviour. Although this behaviour is commonly observed in many steels, deviations from Masing behaviour have also been reported in the literature, with some materials exhibiting a critical strain range beyond which this assumption is no longer valid [26]. The proposed method avoids iterative numerical optimization altogether, relying instead on an analytical fit of the ascending branch that can be implemented in a standard spreadsheet or a few lines of code. The proposed procedure is particularly effective for strain-controlled cyclic loading, especially when the strain range is close to that used for parameter calibration. Furthermore, the estimated parameters can be obtained from tests conducted at a strain ratio equals to −1 or at other strain ratios. These parameters may also be suitable for ratcheting and multiaxial simulations. However, ratcheting modelling is more challenging because the resulting cycles are characterized by a progressively increasing mean strain and may exhibit a strong dependance upon their potentially varying strain range. The accuracy with which the model reproduces the characteristic quantities of these tests depends strongly on the strain range of the cycles considered. As discussed in Section 3, cycles with smaller strain ranges are not always reproduced accurately. Therefore, when accurate ratcheting predictions are required, the proposed estimation procedure can be applied to an hysteresis cycle with a smaller strain range, more representative of the cycles developing during ratcheting.

The method is applied and validated on two low-alloy steels of significant industrial relevance: a 1Cr-1Mo-0.25V rotor steel typically employed in steam turbines [27,28,29,30], and a 42CrMo4+QT steel, a low-alloy steel usually employed in components subjected to heavy cyclic loading and widely investigated in the literature [25,31,32,33,34,35,36,37]. First, the method will be applied to 42CrMo4+QT steel tested at room temperature using two different values of average total strain and four different strain range values. Next, the method will be tested on 1Cr-1Mo-0.25V steel at various temperatures to evaluate its effectiveness, again using four strain range values for each temperature. It will be shown in Section 3 that with only the mid-life and the initial hysteresis cycle for a given strain range, it is possible to estimate the Chaboche–Voce material model parameters for a given temperature and for different loading conditions.

The paper is structured as follows: Section 2 describes the materials investigated and the experimental procedures and presents the constitutive laws adopted and the various steps leading to the derivation of the analytical fitting function. Section 3 presents the results obtained by applying the proposed methodology to the investigated materials over different strain ranges and temperatures, and Section 4 discusses the limitations of the proposed method and outlines possible directions for future development. Finally, Section 5 summarises the main conclusions of the study.

## 2. Materials and Methods

### 2.1. Materials

The first material is 42CrMo4+QT, where “+QT” refers to the quenched and tempered condition. This steel is typically employed in automotive, aircraft and energy sectors for the manufacturing of shafts, gears and connecting components. The cyclic plastic data were taken from [38] while the chemical composition, reported in Table 1, was taken from [39]. A part of the tests was performed in strain control with a strain ratio $R_{\varepsilon }=-1$ (i.e., $\varepsilon_{m}=0$), while the other part was performed at $\varepsilon_{m}=0.50\%$. All the tests were conducted at room temperature (RT). The experimental cycles included here are not all of those available in the reference: a few representative cycles with different hysteresis areas were selected.

The second material investigated is 1Cr-1Mo-0.25V, a martensitic steel commonly used for turbine rotors and designed to operate at temperatures up to 560 °C. Its chemical composition is reported in Table 2. In contrast to Ref. [27], which investigated the fatigue and creep-fatigue behaviour of this material at 500 °C, the present work considers a wider range of temperatures, namely 20 °C, 200 °C, and 400 °C. With regard to the experimental data at 500 °C, only a few representative cycles from the previous study were selected to demonstrate the effectiveness of the methodology proposed in Section 3. All tests were conducted in accordance with ASTM E606/E606M-12 [40] using a Schenk servo-hydraulic testing machine with a maximum load capacity of 100 kN and a high-temperature extensometer with a 25.4 mm gauge length. The tests are strain-controlled and the strains were measured in the longitudinal axis. Further details of the experimental campaign and the geometry of the specimen employed are reported in Table 3.

### 2.2. Methods

#### 2.2.1. Chaboche–Voce Cyclic Plasticity

The cyclic plasticity model adopted in this work is the Chaboche–Voce model, which accounts for both nonlinear kinematic and isotropic hardening. Adopting a Von-Mises yield criterion, for a material subjected to uniaxial tension–compression loading, the yield surface equation reduces to Equation (1):

(1) $$f=\sigma -\chi_{\mathrm{tot}}-R-\sigma_{y,0}=0.$$

**Figure 1:**
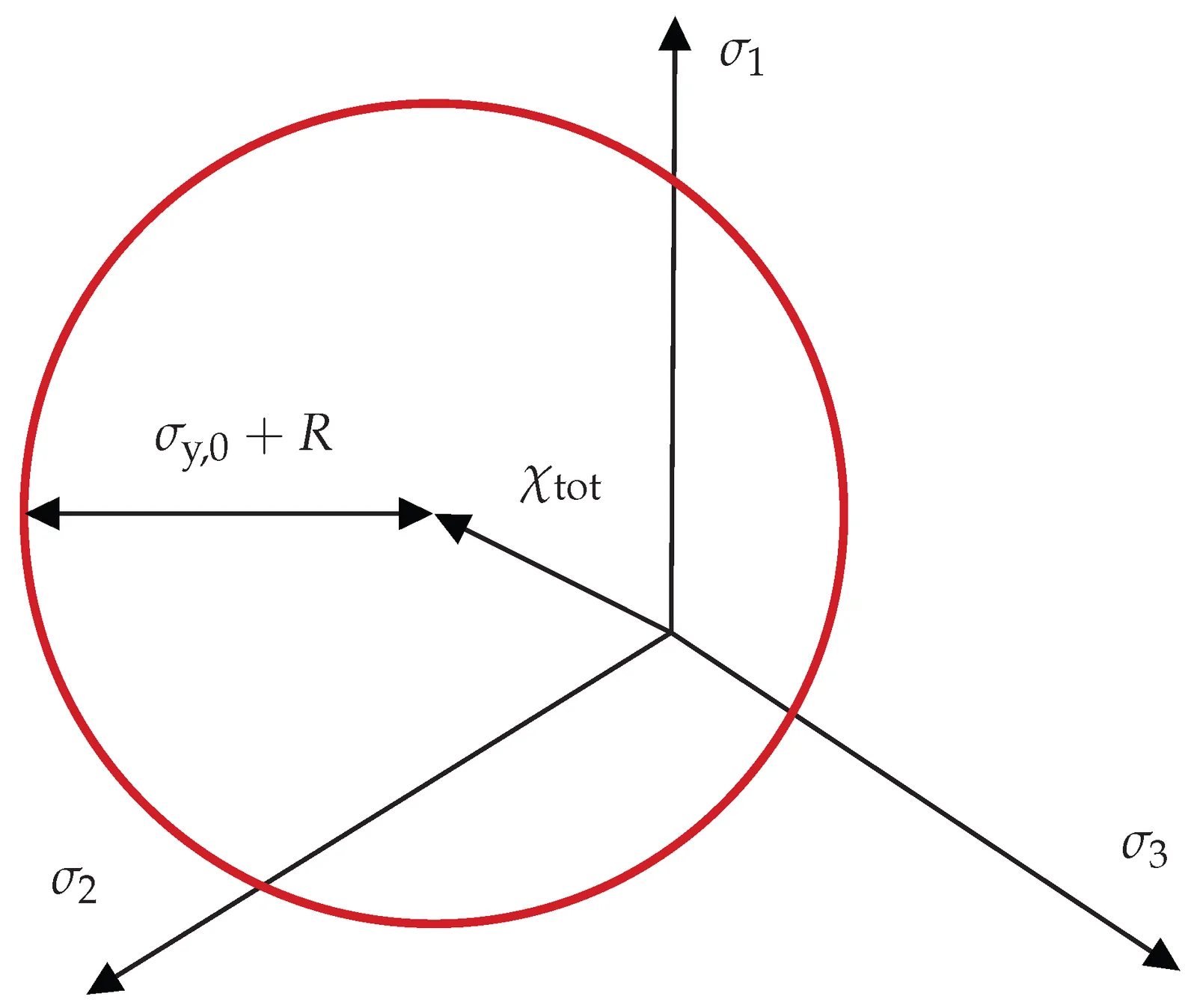
Representation of the yield surface with the kinematic and isotropic hardening components.

In Equation (1), σ denotes the applied stress, $\chi_{\mathrm{tot}}$ is the total backstress, in this case defined as the sum of three backstresses, *R* is the isotropic hardening component described by the Voce law and $\sigma_{y,0}$ is the initial yield stress. A representation of the yield surface with the mentioned hardening components is depicted in Figure 1.

The evolutions of each individual backstress, $\chi_{i}$, the definition of the total backstress, $\chi_{\mathrm{tot}}$, and of the isotropic variable, *R*, are reported, respectively, in Equation (2), Equation (3) and Equation (4):

(2) $$\chi_{\mathrm{tot}}=\sum_{i=1}^{3}\chi_{i}$$

(3) $${\dot{\chi }}_{i}=C_{i}{\dot{\varepsilon }}_{\mathrm{pl}}-\gamma_{i}\chi_{i}\dot{p}$$

(4) $$\dot{R}=b\left(Q-R\right)\dot{p}$$

where ${\dot{\varepsilon }}_{\mathrm{pl}}$ is the plastic strain rate and $\dot{p}$ is the accumulated plastic strain rate. The accumulated plastic strain, *p*, is defined in Equation (5):

(5) $$p=\int |d\varepsilon_{\mathrm{pl}}|.$$

$C_{i}$ and $\gamma_{i}$ are the material parameter of each Chaboche backstress and *b* and *Q* are the Voce model parameters. The numerical values of these parameters for the two materials investigated can be found in Table 4 and Table 5. All the parameters are material- and temperature-dependent. Equation (3) can be integrated assuming a constant plastic strain rate, an arbitrary initial value of the backstress, $\chi_{i,0}$, and of the plastic strain, $\varepsilon_{\mathrm{pl},0}$. The resulting analytical expression, for an ascending ramp (i.e., $\varepsilon_{\mathrm{pl}}-\varepsilon_{\mathrm{pl},0}>0$), is given in Equation (6).

(6) $$\chi_{i}\left(\varepsilon_{\mathrm{pl}}\right)=\frac{C_{i}}{\gamma_{i}}+\left(\chi_{i,0}-\frac{C_{i}}{\gamma_{i}}\right)e^{-\gamma_{i}\left(\varepsilon_{\mathrm{pl}}-\varepsilon_{\mathrm{pl},0}\right)}.$$

The maximum and minimum values of the backstress attained during the stabilized cycle are well established in the literature [2,25] and are given by Equation (7):

(7) $$\chi_{i,\mathrm{stab}}^{\min}=-\frac{C_{i}}{\gamma_{i}}\tanh\left(\frac{\gamma_{i}\Delta \varepsilon_{\mathrm{pl}}}{2}\right)=-\chi_{i,\mathrm{stab}}^{\max}$$

where $\Delta \varepsilon_{\mathrm{pl}}$ is the plastic strain range of the given stabilized cycle. Therefore, considering the ascending branch of the stabilized cycle, from the minimum to the maximum stress, the initial value of the total backstress, $\chi_{\mathrm{tot},\mathrm{stab}}^{\min}$, is given by the sum of the three minimum stabilized backstresses $\chi_{i,\mathrm{stab}}^{\min}$, as shown in Equation (8):

(8) $$\chi_{\mathrm{tot},\mathrm{stab}}^{\min}=\sum_{i=1}^{3}\chi_{i,\mathrm{stab}}^{\min}.$$

Given that a stabilized cycle is defined as a cycle whose response remains constant or varies within an acceptable tolerance, the minimum stabilized backstress value can be assumed as the initial condition for Equation (6). By substituting Equation (7) for $\chi_{i,0}$ into Equation (6) and simplifying, the following expression is obtained for the evolution of the backstress along the ascending branch of the stabilized cycle:

(9) $$\chi_{i}\left(\varepsilon_{\mathrm{pl}}\right)=\frac{C_{i}}{\gamma_{i}}\left[1-\left(1+\tanh\left(\frac{\gamma_{i}\Delta \varepsilon_{\mathrm{pl}}}{2}\right)\right)e^{-\gamma_{i}\left(\varepsilon_{\mathrm{pl}}-\varepsilon_{\mathrm{pl},0}\right)}\right]$$

where $\Delta \varepsilon_{\mathrm{pl}}$ and $\varepsilon_{\mathrm{pl},0}$ are known. At the stabilized cycle the yield stress have reached its saturated value $k=\sigma_{y}\left(p\to \infty \right)$, and is constant. Once replaced in Equation (2) the value of $\chi_{i}$ found in Equation (9), and assumed the saturation of the yield stress (i.e., $\sigma_{y,0}+R=k$), the value of the stress as a function of the plastic strain can be derived from Equation (1) as follows:

(10) $$\sigma \left(\varepsilon_{\mathrm{pl}}\right)=\sum_{i=1}^{3}\frac{C_{i}}{\gamma_{i}}\left[1-\left(1+\tanh\left(\frac{\gamma_{i}\Delta \varepsilon_{\mathrm{pl}}}{2}\right)\right)e^{-\gamma_{i}\left(\varepsilon_{\mathrm{pl}}-\varepsilon_{\mathrm{pl},0}\right)}\right]+k.$$

**Figure 2:**
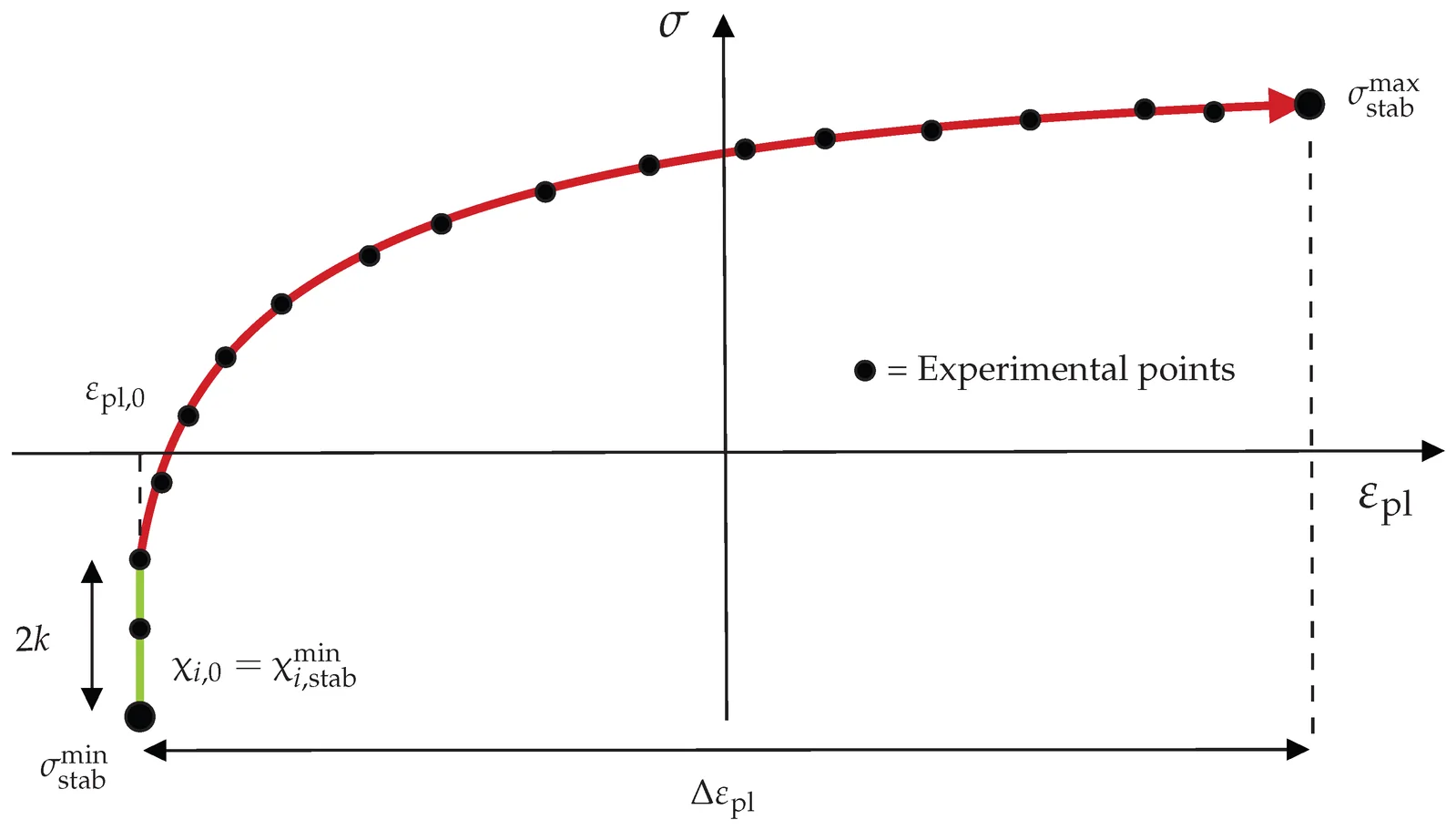
Representation of ascending plastic branch fitted. The black dots represent the experimental data.

Equation (10), for a given stabilized cycle, represents an analytical equation in terms of 7 unknown parameters in the $\sigma -\varepsilon_{\mathrm{pl}}$ plane. The value of *k* itself is not known a priori and must be determined through the fitting procedure. Although the initial part of the stabilized cycle—idealized by a vertical segment in the $\sigma -\varepsilon_{\mathrm{pl}}$ plane—belongs to the elastic region, it must still be considered in the fitting since the value of *k* is unknown. Once the initial fit has been performed and the value of *k* has been determined, the elastic region can be subtracted and the fitting procedure repeated, thereby removing *k* from the analytical expression. This second fitting step provides a more accurate representation of the experimental data, particularly of the tangent modulus in the vicinity of the tensile stress peak. In Figure 2, the green portion represents the elastic region, while the red portion represents the plastic region described by Equation (10).

Regarding the number of backstresses, as mentioned earlier, they are assumed to be 3 and $\gamma_{3}$ has been assumed to be equal to one in order to provide a slightly linear backstress component for better capturing the tangent modulus at the tensile peak stress of the cycle. This choice can be found also in [25,41]. The number of parameters to be evaluated with the fitting of Equation (10) reduces to 6.

With regard to the parameters of Voce’s isotropic hardening model, once $C_{1}$, $\gamma_{1}$, $C_{2}$, $\gamma_{2}$, $C_{3}$, and *k* have been determined ($\gamma_{3}=1$), the parameter *Q* can be directly obtained from the first hysteresis cycle. Considering the ascending branch of the first cycle, which starts from the origin and therefore assumes zero initial plastic strain and zero backstress, the corresponding expression reduces to Equation (11):

(11) $$\chi_{i}\left(\varepsilon_{\mathrm{pl}}\right)=\frac{C_{i}}{\gamma_{i}}\left(1-e^{-\gamma_{i}\varepsilon_{\mathrm{pl}}}\right).$$

The maximum value reached by the generic backstress $\chi_{i}$ during the first cycle can be obtained by evaluating the corresponding expression at the maximum plastic strain reached, $\varepsilon_{\mathrm{pl},1}^{\max}$. Since the saturated yield stress *k* is defined as the sum of the initial yield stress, $\sigma_{y,0}$, and the isotropic hardening parameter *Q*, the latter can be related to the initial yield stress as $\sigma_{y,0}=k-Q$. Therefore, considering the peak stress in tension reached during the first cycle, $\sigma_{1}^{\max}$, and employing Equation (1), the following equation can be written:

(12) $$\sigma_{1}^{\max}=\sum_{i=1}^{3}\frac{C_{i}}{\gamma_{i}}\left(1-e^{-\gamma_{i}\varepsilon_{\mathrm{pl},1}^{\max}}\right)+k-Q.$$

Solving Equation (12) for *Q* allows us to determine its numerical value. For simplicity, since the plastic strain accumulated along the branch leading to the first tensile peak is small, the effect of isotropic hardening is neglected. The last parameter to be found, *b*, can be obtained simply by using the equation given in Refs. [1,3],

(13) $$\frac{{\sigma_{N}}^{\max}-\sigma_{1}^{\max}}{\sigma_{\mathrm{stab}}^{\max}-\sigma_{1}^{\max}}=\frac{R}{Q}=1-e^{-bp},$$

where ${\sigma_{N}}^{\max}$ is the value of the peak stress in tension for a generic cycle N from the first to the stabilized cycle. Fitting experimental data regarding the decrease, or increase, in the peak stress during cycling with Equation (13), the value of *b* is obtained. The value of the accumulated plastic strain with N cycles can be easily estimated with $p=2N\Delta \varepsilon_{\mathrm{pl},\mathrm{stab}}$, where $\Delta \varepsilon_{\mathrm{pl},\mathrm{stab}}$ is the plastic strain range at the stabilized cycle. Otherwise, *p* can be calculated manually if all hysteresis cycles are available, or by interpolation if they are available at a certain sampling frequency.

In conclusion, by applying Equation (10) to the half-life cycle, Equation (12) to the first cycle of the same test, and Equation (13) to the cycles spanning from the first to the stabilized cycle, the parameters of the Chaboche–Voce model can be determined for a given material and temperature. In the following, the proposed procedure is applied to experimental tests with $R_{\varepsilon }=-1$; however, the methodology is also applicable to tests with arbitrary strain ratios. The cycle selected for the evaluation of the Chaboche parameters is the one corresponding to the largest Δε, as discussed in Section 3.

#### 2.2.2. Isotropic Hardening Through Variable-γ Formulation

In some cases, Voce’s isotropic hardening model may not be the most suitable for modeling cycles prior to stabilization, even though it remains the simplest and most intuitive model, based on the expansion or contraction of the elastic region. Another possible approach to modelling isotropic hardening is to modify the kinematic hardening law by introducing a multiplicative factor, dependent on the accumulated plastic strain, *p*, that scales the parameter γ. In this formulation, the evolution equation for a generic backstress is given by [1,3] (Equation (14)):

(14) $${\dot{\chi }}_{i}=C_{i}{\dot{\varepsilon }}_{\mathrm{pl}}-\Phi_{i}\left(p\right)\gamma_{i}\chi_{i}\dot{p}$$

where $\Phi_{i}\left(p\right)$ is the variable responsible for the variation in $\gamma_{i}$. The evolution of the $\Phi_{i}\left(p\right)$ with *p* is described by the following relation (Equation (15)),

(15) $${\dot{\Phi }}_{i}=\omega \left(\Phi_{\infty }-\Phi \right)\dot{p},$$

which is similar to the Voce law evolution for $\dot{R}$ (Equation (4)). At first glance, this formulation introduces two additional temperature-dependent parameters for each backstress, increasing the total number of parameters to be estimated for isotropic hardening from two to six. To avoid increasing the number of parameters relative to the formulation based on Voce’s law, only the γ parameter associated with a single backstress was allowed to vary, specifically that corresponding to the highest γ value. In the case of cyclic softening, for example, the product $\Phi_{i}\gamma_{i}$ is expected to be higher at the stabilized cycle than in the first cycle, since higher values of $\gamma_{i}$ not only increase the rate of exponential saturation but also lead to lower backstress values and, consequently, lower stress levels.

To determine the parameters of this modified formulation, the variation is introduced only for one specific backstress, e.g., backstress 1. Once all the parameters have been estimated from the stabilized cycle, the following equation can be formulated by considering, as before, the peak tensile stress reached during the first cycle:

(16) $$\sigma_{1}^{\max}=\frac{C_{1}}{\gamma_{1,0}}\left(1-e^{-\gamma_{1,0}\varepsilon_{\mathrm{pl},1}^{\max}}\right)+\sum_{i=2,3}\frac{C_{i}}{\gamma_{i}}\left(1-e^{-\gamma_{i}\varepsilon_{\mathrm{pl},1}^{\max}}\right)+k.$$

In Equation (16) $\gamma_{1,0}$ is the only unknown and represents the value of the $\gamma_{1}\left(p\right)$ at the first cycle (i.e., p=0), since all other $C_{i}$ and $\gamma_{i}$ values are those estimated from the stabilized cycle. Once $\gamma_{1,0}$ is determined by solving the resulting non-linear equation, and noting that $\gamma_{1}$ in the stabilized cycle is equal to $\Phi_{1,\infty }\left(p\right)\gamma_{1,0}$, the corresponding value of can be obtained as follows:

(17) $$\Phi_{1,\infty }\left(p\right)=\frac{\gamma_{1}}{\gamma_{1,0}}.$$

If the value of $\Phi_{i,\infty }$ defined in Equation (17) is greater than 1, cyclic softening occurs, whereas values of $\Phi_{i,\infty }$ between 0 and 1 correspond to cyclic hardening. Finally, the value of ω of Equation (15) can be obtained from Ref. [1] from fitting the evolution of the peak stress with *p* similarly for the parameter *b* of Voce (Equation (18)):

(18) $$\frac{{\sigma_{N}}^{\max}-\sigma_{1}^{\max}}{\sigma_{\mathrm{stab}}^{\max}-\sigma_{1}^{\max}}=\frac{\frac{1}{\Phi_{1}\left(p\right)}\tanh\left(\frac{\gamma_{1,0}\Phi_{1}\left(p\right)\Delta \varepsilon_{\mathrm{pl}}}{2}\right)-\tanh\left(\frac{\gamma_{1,0}\Delta \varepsilon_{\mathrm{pl}}}{2}\right)}{\frac{1}{\Phi_{1,\infty }}\tanh\left(\frac{\gamma_{1}\Delta \varepsilon_{\mathrm{pl}}}{2}\right)-\tanh\left(\frac{\gamma_{1,0}\Delta \varepsilon_{\mathrm{pl}}}{2}\right)}.$$

The comparison between Voce and this formulation is provided in Section 3.2 for the 1Cr-1Mo-0.25V steel at 500 °C.

## 3. Results

### 3.1. Result with Chaboche–Voce Model

**Figure 3:**
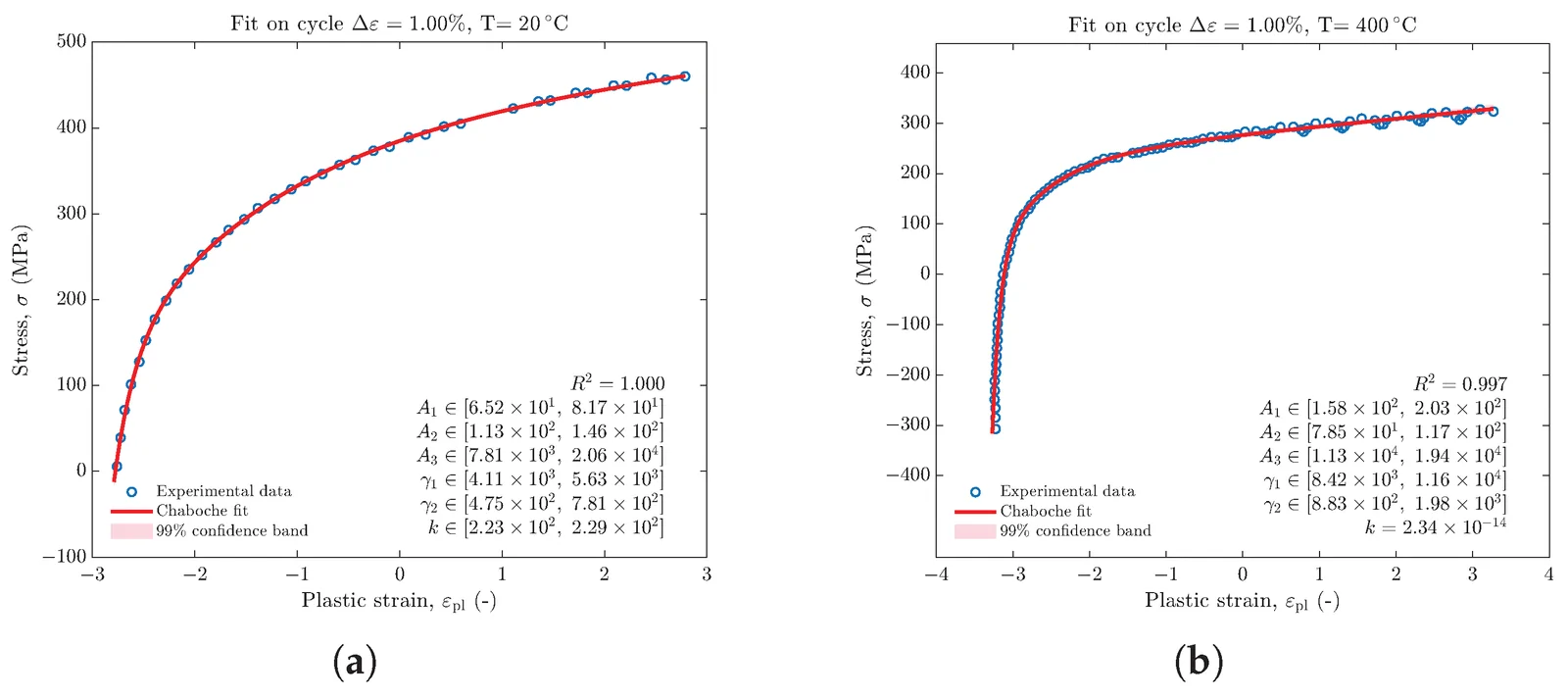
Comparison between the experimental data and the fitted function for the two tests with the largest strain ranges for 1Cr-1Mo-0.25V at 20 °C (**a**) and 400 °C (**b**). The confidence intervals for the estimated parameters ($\gamma_{3}=1$) are reported in the lower panels, while the light-red shaded area in the plot represents the 99% confidence interval.

In this section the results of the application of the method presented in Section 2.2.1 are presented. First of all, the Young modulus is calculated by fitting the tensile unloading at the midlife cycle of the stabilized cycle at the maximum Δε. The fitting procedure was carried out with a non-linear least-squares procedure based on a trust-region algorithm and the results of the fitting procedure are shown in Figure 3 for two cases representative of the campaign with a 99% confidence interval. The analytical function given in Equation (10) has been reformulated by fitting the parameter $A_{i}$ instead of $C_{i}$, where $A_{i}=C_{i}/\gamma_{i}$. This choice was made to bring the orders of magnitude of the parameters $A_{i}$ and $\gamma_{i}$ closer together, thereby facilitating the fitting procedure. The fit was carried out in two steps: a first fit on the whole branch provided an estimate of the yield stress *k*. The elastic reloading portion of the branch was then excluded, and the fit was repeated on the remaining data to obtain the final parameters.

The 99% confidence intervals of the parameters were computed from the Jacobian of the model at the solution, using a linearized estimate of the covariance matrix and Student’s *t* distribution. The corresponding confidence band on the fitted curve was obtained by propagating the same covariance matrix to the model prediction. The goodness of fit was quantified through the coefficient of determination R^2^. The puntual value of *k* is reported in Figure 3b because no confidence interval for this parameter was found by the algorithm. In addition, the confidence interval limits for each parameter are provided. In both cases, the coefficient of determination, R^2^, is equal to one, and the confidence intervals are extremely narrow. The numerical values of the fitted parameters can be found in Table 4 for 42CrMo4+QT and in Table 5 for the 1Cr-1Mo-0.25V steel.

**Figure 4:**
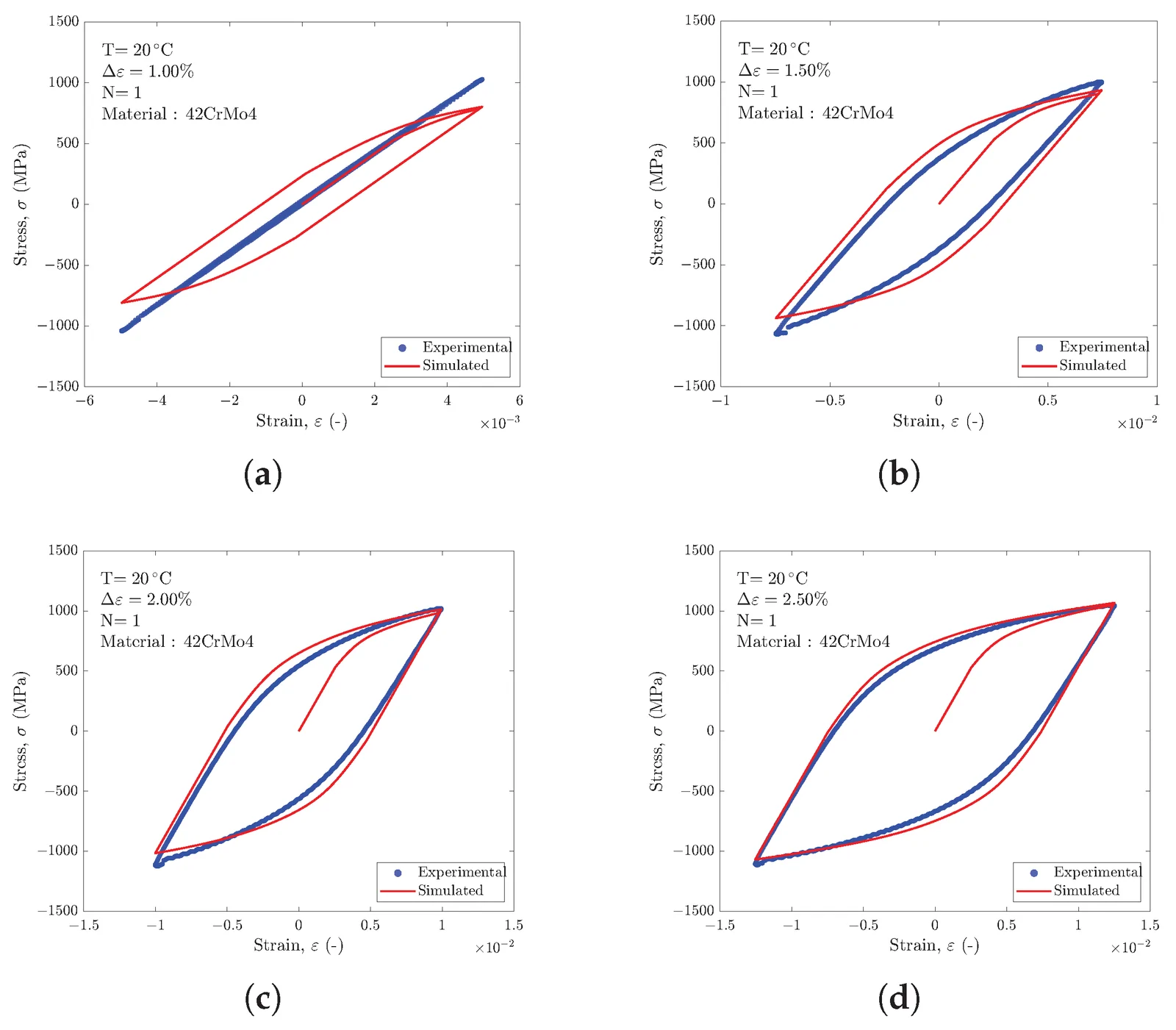
Numerically simulated and experimental initial (N = 1) cycles for material 42CrMo4+QT at T = 20 °C and at Δε=1.00% (**a**), Δε=1.50% (**b**), Δε=2.00% (**c**) and Δε=2.50% (**d**).

**Figure 5:**
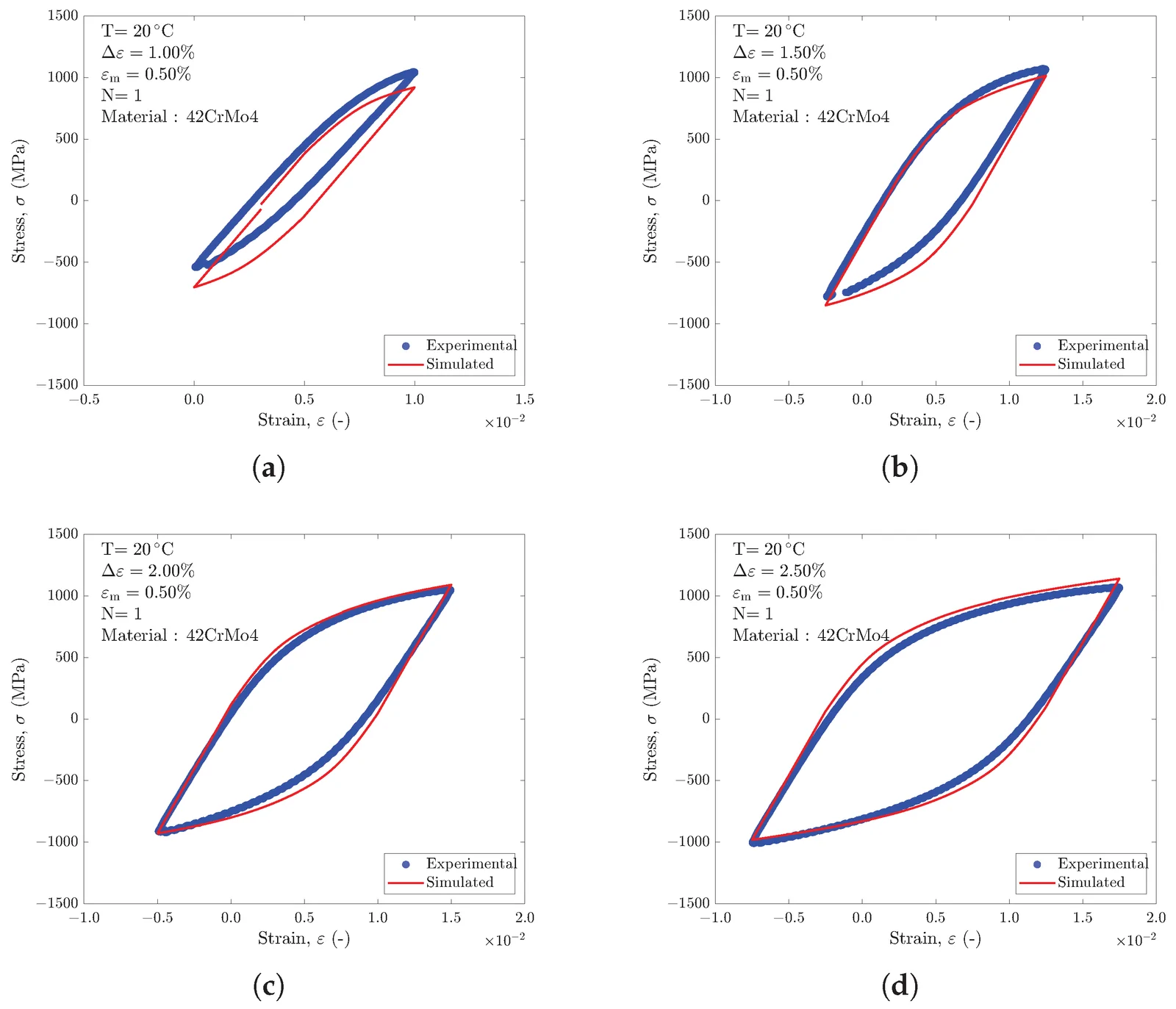
Numerically simulated and experimental initial (N =1) cycles for material 42CrMo4+QT at T =20 °C with a mean strain of $\varepsilon_{m}=0.50\%$ and at Δε=1.00% (**a**), Δε=1.50% (**b**), Δε=2.00% (**c**) and Δε=2.50% (**d**).

**Figure 6:**
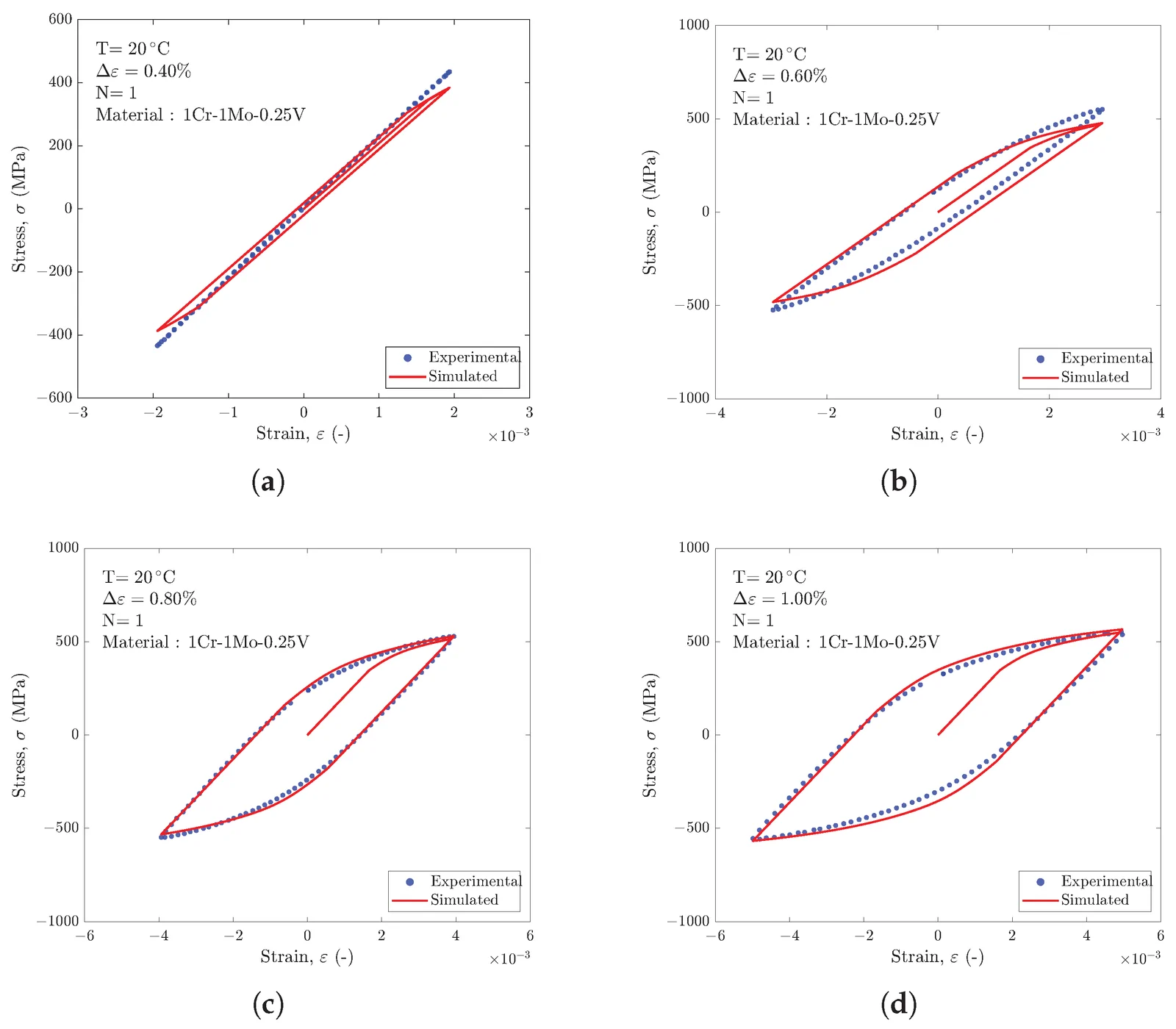
Numerically simulated and experimental initial cycles (N =1) for material 1Cr-1Mo-0.25V at T =20 °C and at Δε=0.40% (**a**), Δε=0.60% (**b**), Δε=0.80% (**c**) and Δε=1.00% (**d**).

**Figure 7:**
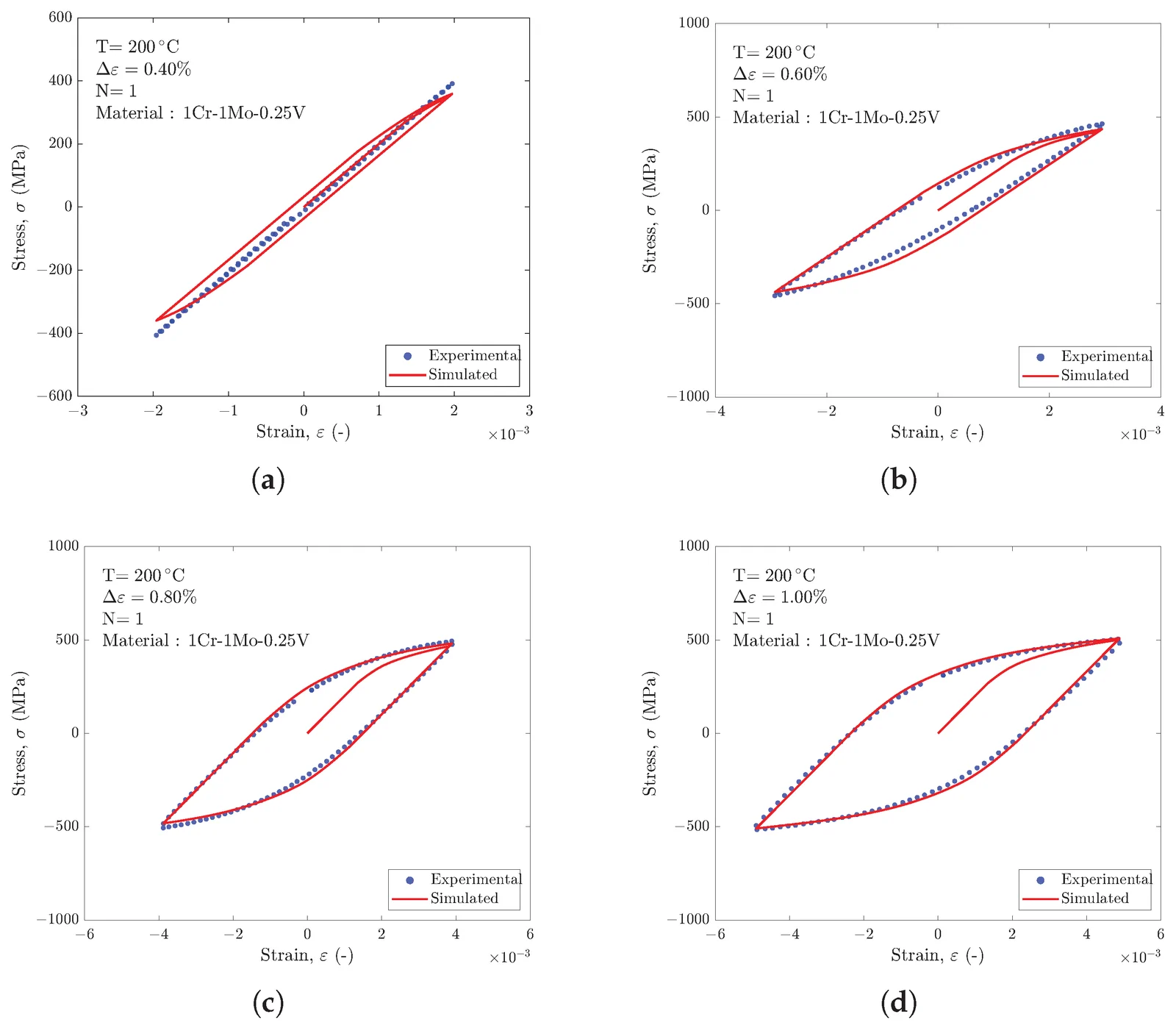
Numerically simulated and experimental initial cycles (N =1) for material 1Cr-1Mo-0.25V at T =200 °C and at Δε=0.40% (**a**), Δε=0.60% (**b**), Δε=0.80% (**c**) and Δε=1.00% (**d**).

**Figure 8:**
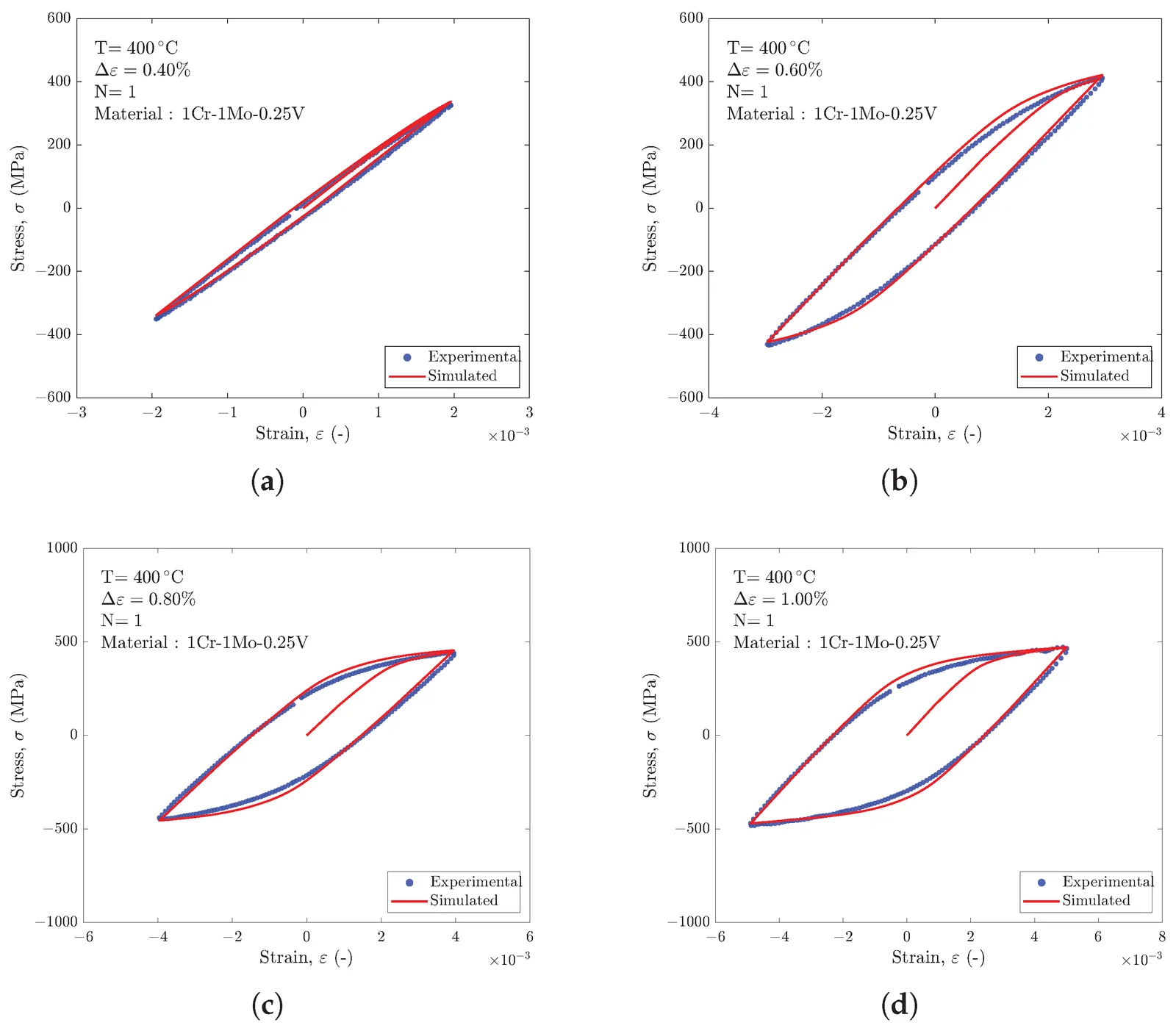
Numerically simulated and experimental initial cycles (N =1) for material 1Cr-1Mo-0.25V at T =400 °C and at Δε=0.40% (**a**), Δε=0.60% (**b**), Δε=0.80% (**c**) and Δε=1.00% (**d**).

**Figure 9:**
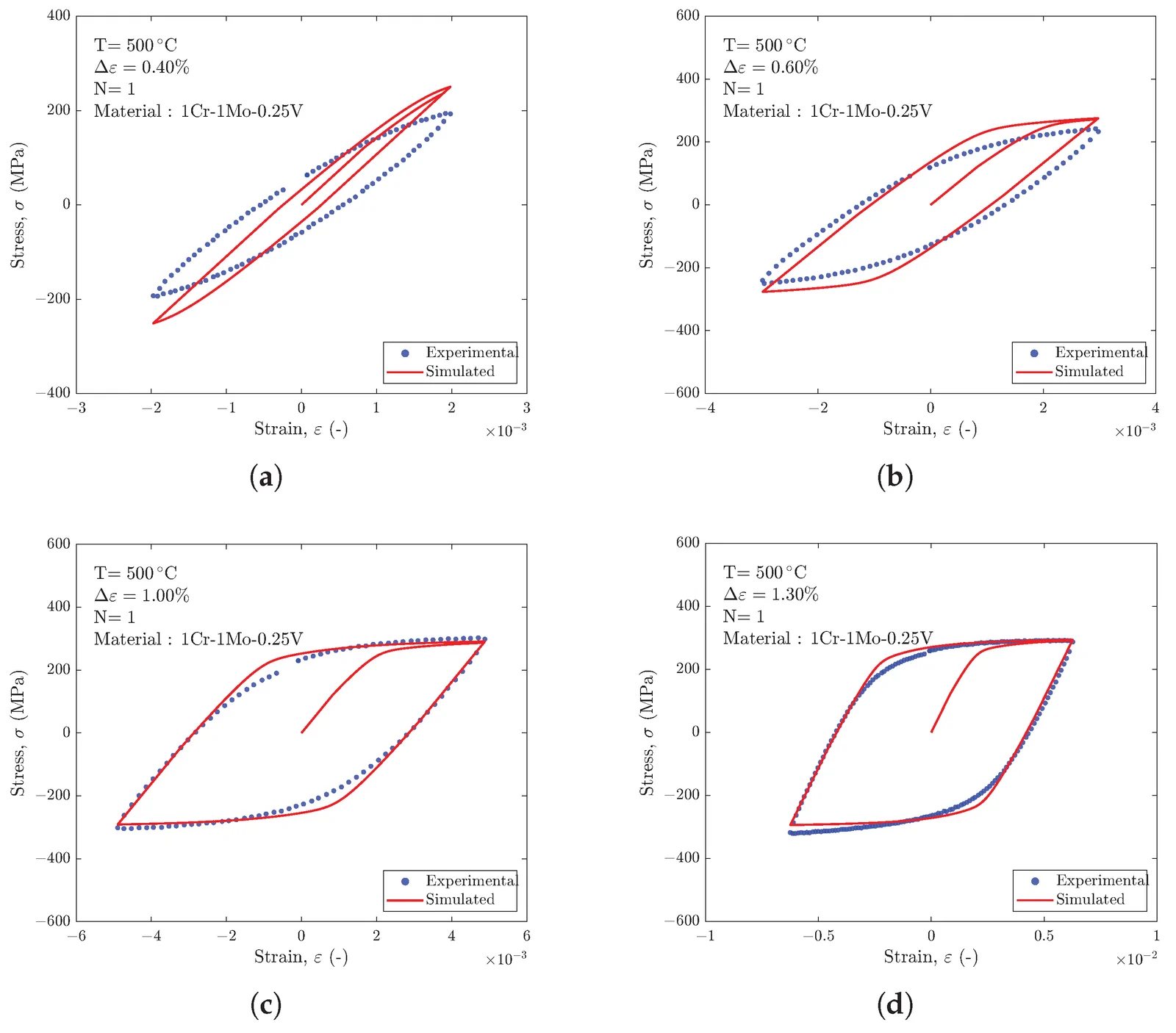
Numerically simulated and experimental initial cycles (N =1) for material 1Cr-1Mo-0.25V at T =500 °C and at Δε=0.40% (**a**), Δε=0.60% (**b**), Δε=1.00% (**c**) and Δε=1.30% (**d**).

**Figure 10:**
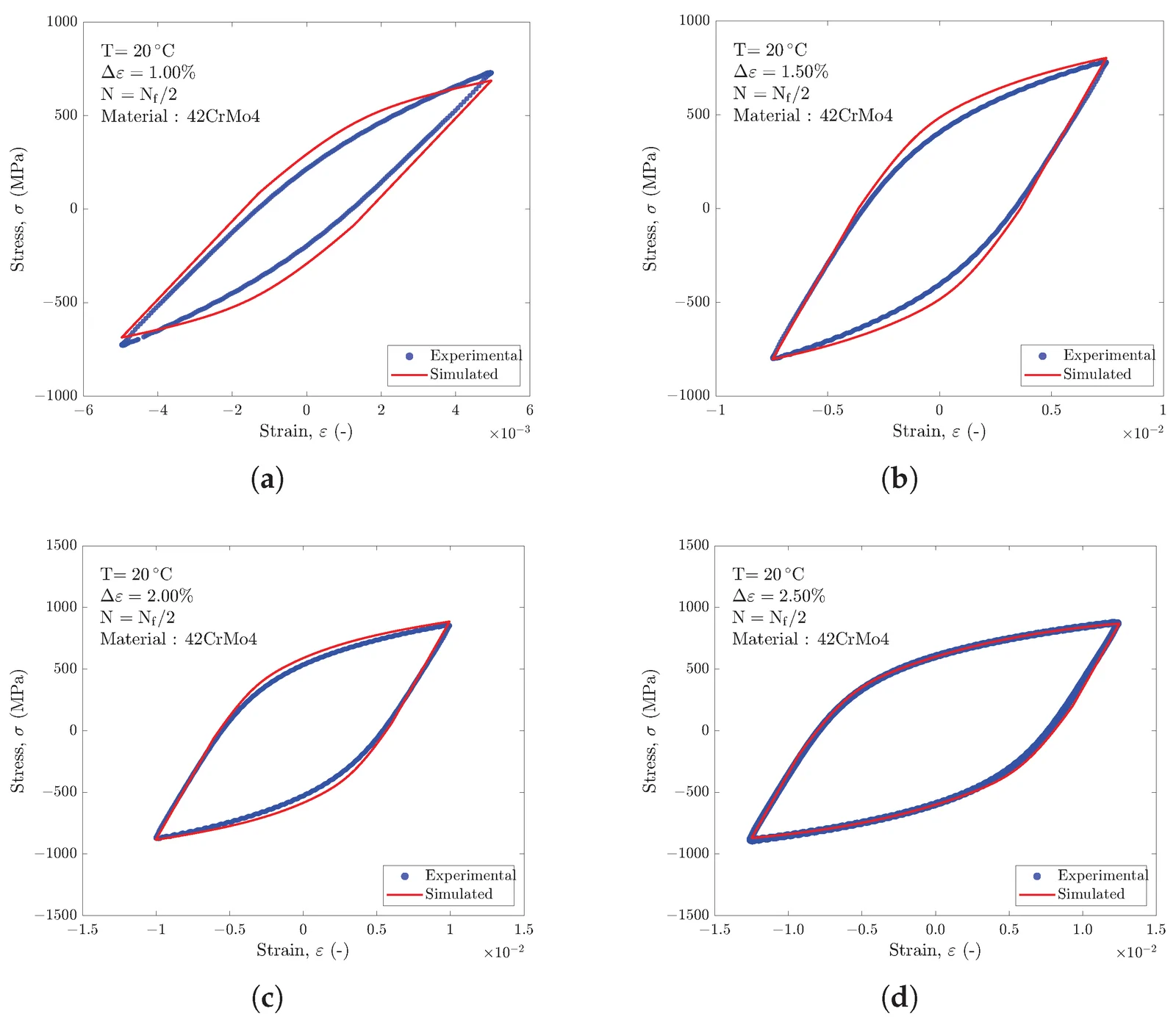
Numerically simulated and experimental midlife cycles (N $=N_{f}/2$) for material 42CrMo4+QT at T = 20 °C and at Δε=1.00% (**a**), Δε=1.50% (**b**), Δε=2.00% (**c**) and Δε=2.50% (**d**).

**Figure 11:**
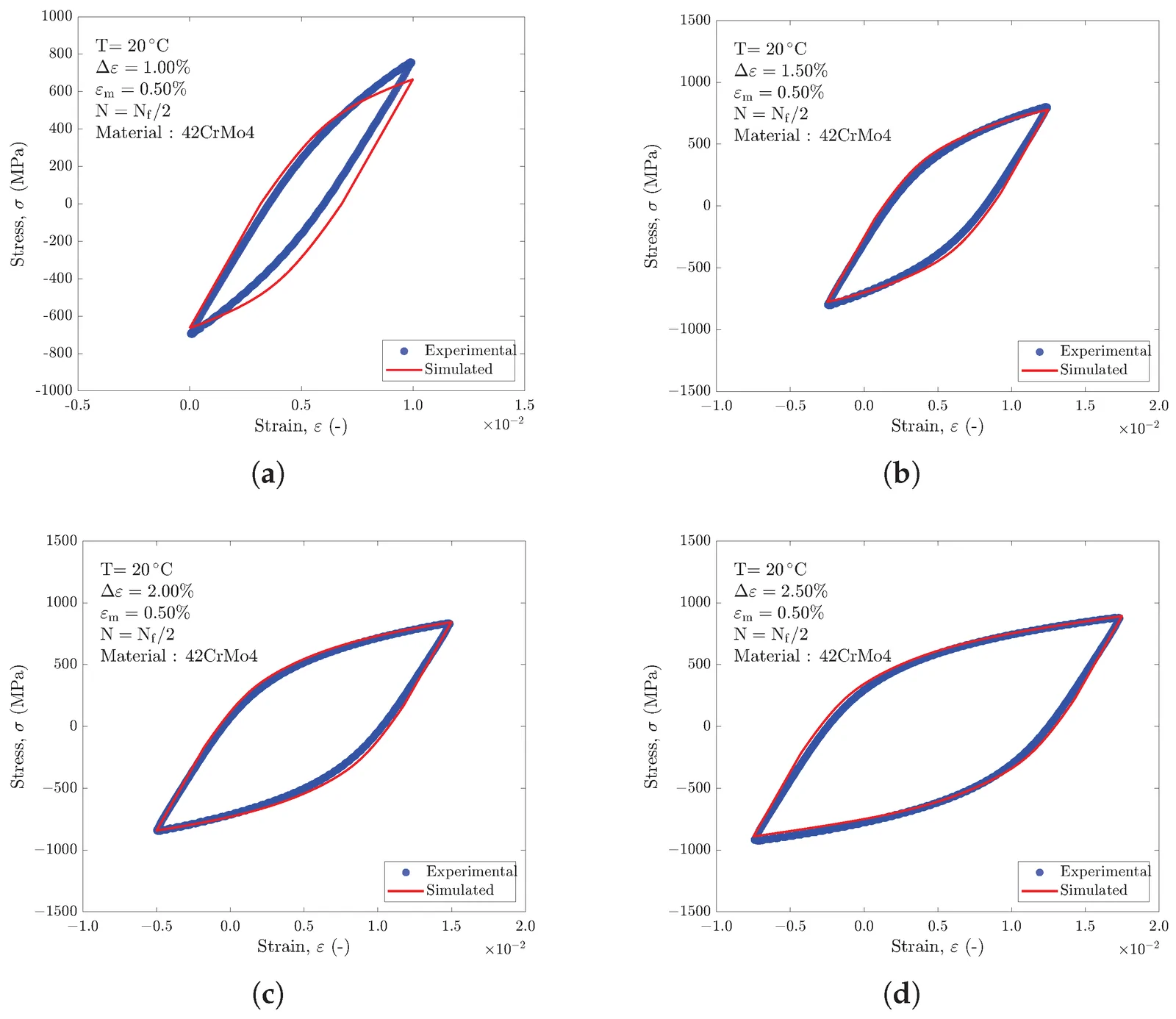
Numerically simulated and experimental midlife cycles (N $=N_{f}/2$) for material 42CrMo4+QT at T =20 °C with a mean strain of $\varepsilon_{m}=0.50\%$ and at Δε=1.00% (**a**), Δε=1.50% (**b**), Δε=2.00% (**c**) and Δε=2.50% (**d**).

**Figure 12:**
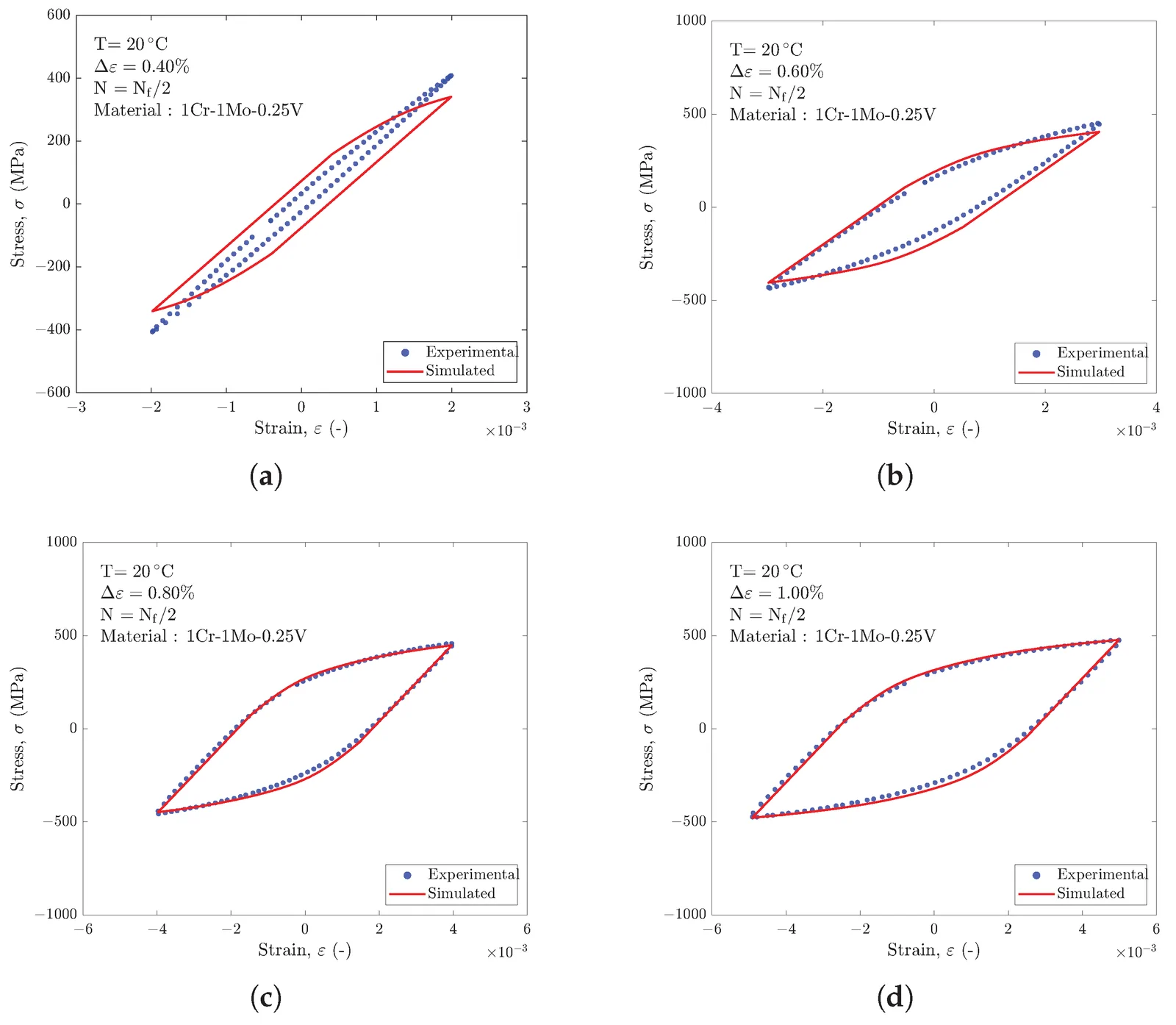
Numerically simulated and experimental midlife cycles (N $=N_{f}/2$) for material 1Cr-1Mo-0.25V at T =20 °C and at Δε=0.40% (**a**), Δε=0.60% (**b**), Δε=0.80% (**c**) and Δε=1.00% (**d**).

**Figure 13:**
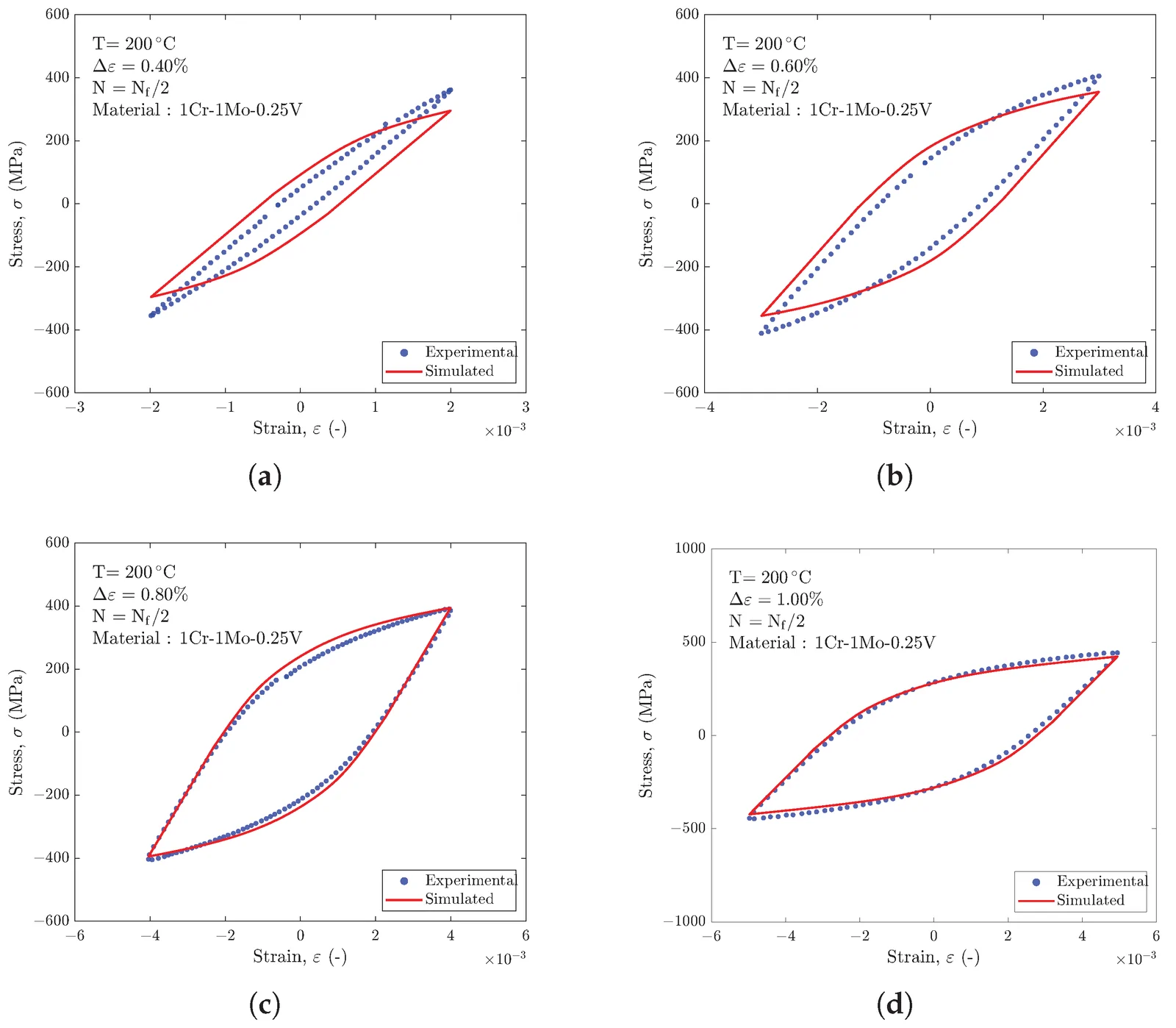
Numerically simulated and experimental midlife cycles (N $=N_{f}/2$) for material 1Cr-1Mo-0.25V at T =200 °C and at Δε=0.40% (**a**), Δε=0.60% (**b**), Δε=0.80% (**c**) and Δε=1.00% (**d**).

**Figure 14:**
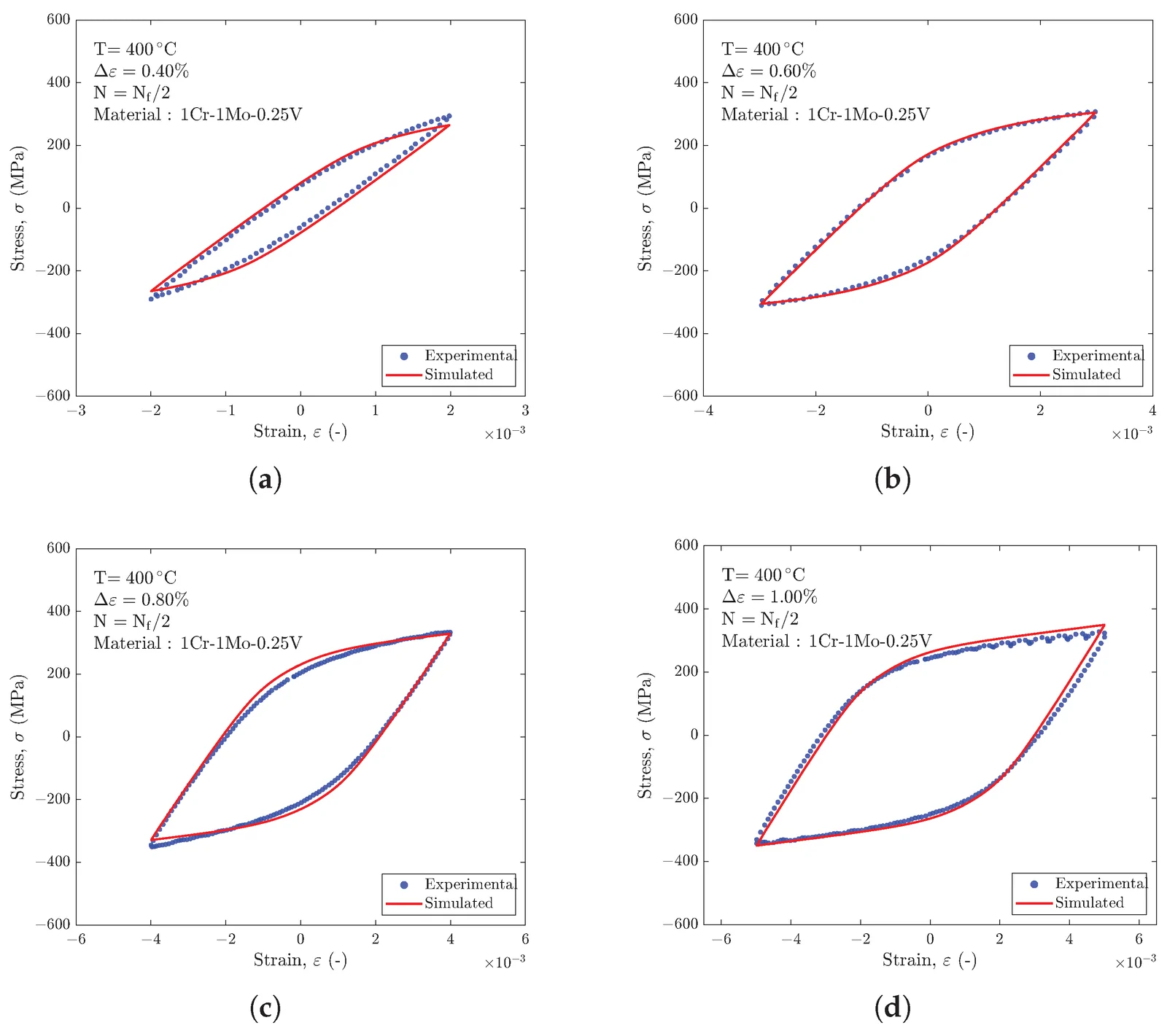
Numerically simulated and experimental midlife cycles (N $=N_{f}/2$) for material 1Cr-1Mo-0.25V at T =400 °C and at Δε=0.40% (**a**), Δε=0.60% (**b**), Δε=0.80% (**c**) and Δε=1.00% (**d**).

**Figure 15:**
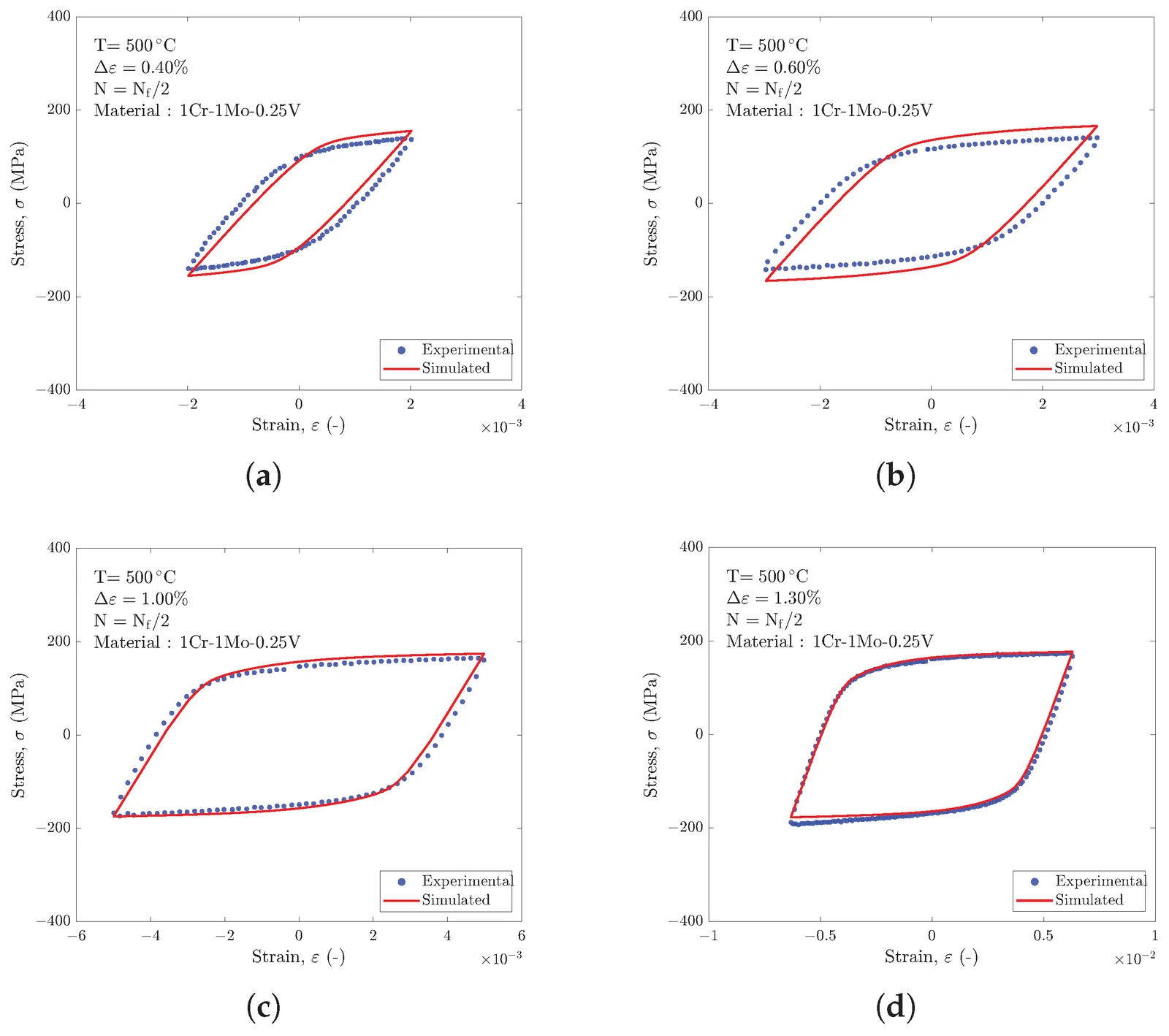
Numerically simulated and experimental midlife cycles (N $=N_{f}/2$) for material 1Cr-1Mo-0.25V at T =500 °C and at Δε=0.40% (**a**), Δε=0.60% (**b**), Δε=1.00% (**c**) and Δε=1.30% (**d**).

The estimated parameters were used to numerically simulate the cyclic plastic behaviour under the different loading and temperature conditions considered. Figure 4, Figure 5, Figure 6, Figure 7, Figure 8 and Figure 9 compare the simulated and experimental initial hysteresis cycles for the 42CrMo4+QT steel at 20 °C with two different values of $\varepsilon_{m}$ as well as for 1Cr-1Mo-0.25V steel at 20 °C, 200 °C, 400 °C, and 500 °C. Instead, Figure 10, Figure 11, Figure 12, Figure 13, Figure 14 and Figure 15 compare simulated and experimental mid-life cycles. For 42CrMo4+QT steel, the parameters were calibrated using the cycles at $R_{\varepsilon }=-1$, and the same parameters were used for the simulations at $\varepsilon_{m}=0.50\%$.

**Figure 16:**
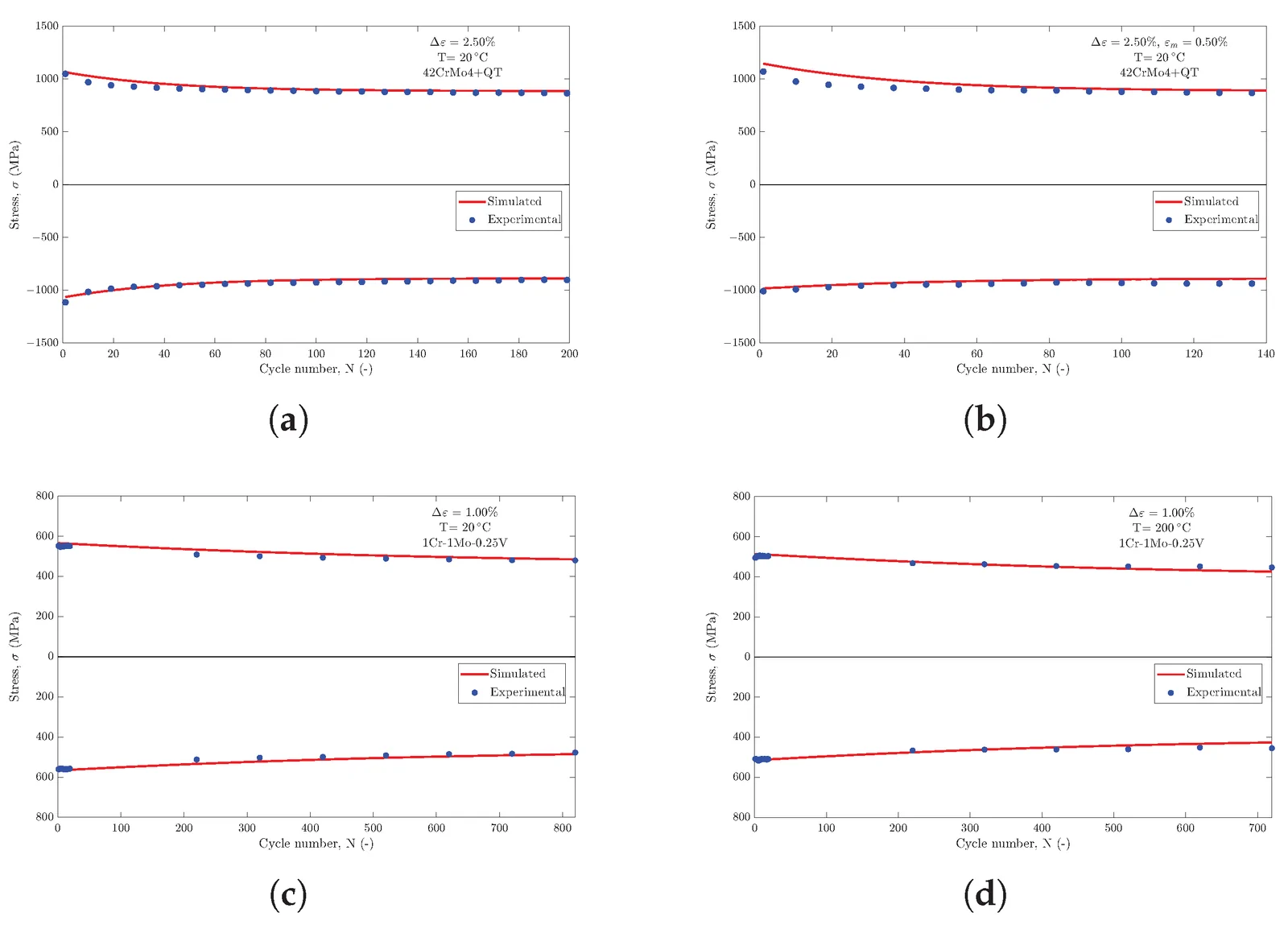

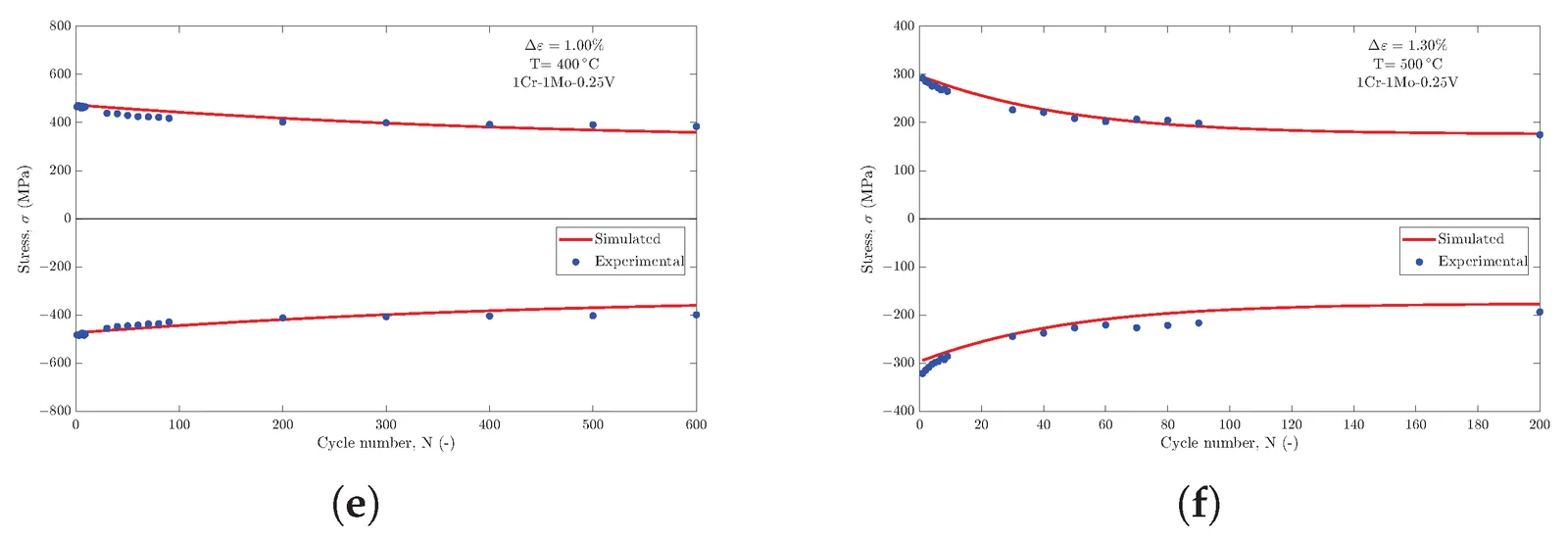
Simulation of the tensile and compressive peak stresses during cyclic loading for the 42CrMo4+QT at two different total mean stress values (**a**,**b**) and for 1Cr-1Mo-0.25V at different temperatures (**c**–**f**). Only the conditions corresponding to the maximum Δε are examined.

Finally, Figure 16 shows the evolution of the tensile and compressive peak stresses during cyclic loading for the five conditions corresponding to the largest strain ranges. It should be noted that the hysteresis cycles shown correspond to the initial and half-life cycles. In some cases, the experimental peak stress does not reach a well-defined saturated value; therefore, the cycles have been consistently identified at $N=N_{f}/2$. In Figure 16, by contrast, the tensile and compressive peak stresses are plotted up to the onset of cyclic stabilization, where observed, whereas the half-life cycle is shown only for the 1Cr-1Mo-0.25V alloy at 500 °C.

### 3.2. Results with Variable-γ Isotropic Hardening

**Figure 17:**
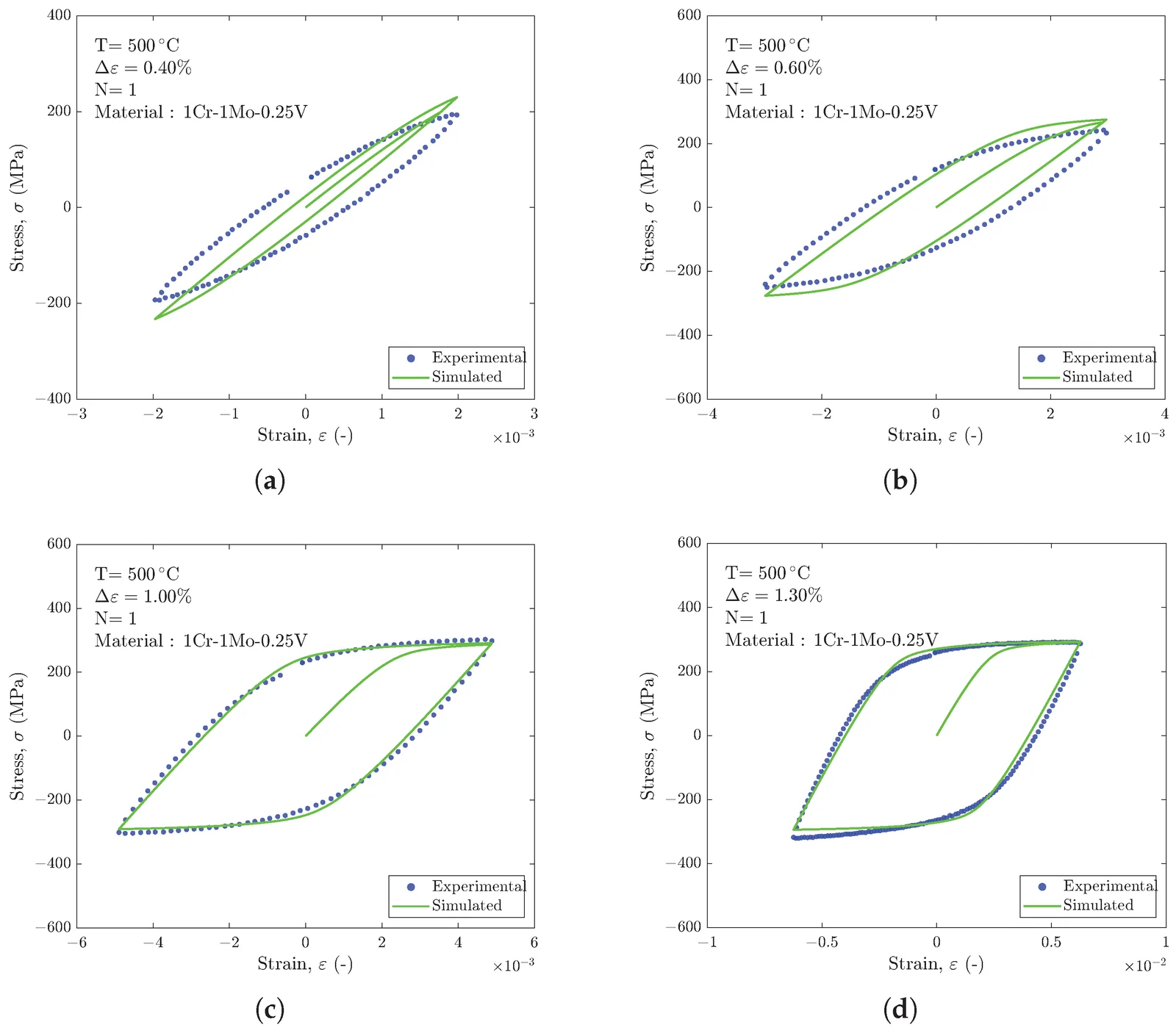
First-cycle response of 1Cr-1Mo-0.25V at 500 °C using the variable-$\gamma_{1}$ formulation at Δε=0.40% (**a**), Δε=0.60% (**b**), Δε=1.00% (**c**) and Δε=1.30% (**d**).

Figure 17 presents the simulations obtained with a variable $\gamma_{1}$ for the test conducted at 500 °C for the material 1Cr-1Mo-0.25V. This figure should be compared with Figure 9. The initial value of the first backstress parameter is found to be $\gamma_{1,0}=3300$, which, as expected, is lower than its half-life value (i.e., $\gamma_{1}=6734$), thereby resulting in a higher stress peak. As a result, from Equation (17), $\Phi_{1,\infty }=1.918$.

With this isotropic hardening formulation, the initial yield stress in the first cycle coincides with *k*, which is practically zero. The most significant improvements obtained with this modeling approach are observed at Δε=1.30% and Δε=1.00%, particularly in the plastic knee region. For the two lower strain ranges, however, no significant improvement is observed.

## 4. Discussion

With regard to the parameters estimated for 1Cr-1Mo-0.25V at the different temperatures (Table 5), it is immediately apparent that, with the exception of $\gamma_{3}=1$, the $\gamma_{i}$ values consistently differ by approximately one order of magnitude.

The temperature dependence of the estimated parameters is also noteworthy. In particular, the saturated yield stress obtained from the fitting procedure is of the order of $10^{2}$ MPa at temperatures below 400 °C. As the temperature approaches the creep regime, however, its value decreases dramatically, becoming practically negligible.

This finding is particularly interesting because it suggests that, at high temperatures, yielding can be considered almost zero, and the apparently linear elastic regions can be accurately reproduced by a sum of exponential functions, as in the Chaboche model, provided that the corresponding hardening rates are appropriately calibrated.

With regard to the isotropic hardening parameters, the value of *Q* remains practically unchanged across the different temperatures, whereas *b* exhibits a much larger variation. The parameter *b* controls the rate at which the material approaches the stabilized cycle (or half-life cycle) as a function of the accumulated plastic strain *p*. It is therefore intuitive that *b* tends towards very small values at low temperatures, where a larger amount of plastic deformation is required to reach the stabilized condition.

Comparing the parameters estimated at 500 °C with those obtained in the previous study [27], where the procedure proposed in Ref. [25] was adopted, no significant differences can be observed in the $C_{i}$ and $\gamma_{i}$ parameters. These results demonstrate that, although the two procedures rely on different evaluation approaches, both provide comparable parameter sets capable of accurately reproducing the experimental hysteresis cycles. This further highlights the non-uniqueness of the parameter-evaluation problem, as different parameter sets may provide comparably accurate representations of the material response [22].

Regarding the temperature dependence of the parameters, further improvements could be achieved in terms of their monotonicity. This aspect is particularly relevant when simulating thermo-mechanical cycles [42,43], for which it is common to account explicitly for the temperature dependence of the backstress evolution through the $C_{i}\left(T\right)$ parameters, as shown, for example, in Equation (19):

(19) $${\dot{\chi }}_{i}=C_{i}{\dot{\varepsilon }}_{\mathrm{pl}}-\gamma_{i}\chi_{i}\dot{p}+\frac{1}{C_{i}}\frac{\partial C_{i}}{\partial T}\chi_{i}\dot{T}.$$

One possible improvement would be to provide the fitting algorithm with the sequence of temperatures considered and use the parameters estimated at one temperature to constrain the fitting at the subsequent or preceding temperature, thereby enforcing a physically consistent trend. Other approaches for constraining the temperature dependence of the Chaboche parameters have also been reported in the literature, such as the use of a Boltzmann-type function, as proposed in [43]. The data reported in Table 5 can be used for thermo-mechanical simulations, although non-monotonic temperature trends in the constitutive parameters are known to potentially cause convergence issues. One possible approach is to use the estimated parameter sets corresponding to the two extreme temperatures of the thermo-mechanical cycle, as in the case of in-phase or out-of-phase loading. Alternatively, the temperature dependence of the estimated parameters can be described using suitable fitting functions, allowing parameter sets to be obtained as a continuous function of temperature without relying on direct interpolation between discrete values. Incorporating prior information on the expected temperature dependence of the parameters and assessing its effectiveness against thermo-mechanical experimental results represent potential directions for future work.

For the tests on 42CrMo4+QT, Figure 10 and Figure 11 show that the parameters estimated from fully reversed cycles ($R_{\varepsilon }=-1$) accurately reproduce the response of cycles with a positive mean strain. For temperature dependence, as only RT data are considered in the present work, no conclusions can be drawn. Nevertheless, the results can be compared with those reported in [25]. Although a different modeling approach was adopted in that study, the saturated yield stress is comparable, with values of 331 MPa in the present work and 398 MPa [25].

For the hysteresis cycles of 1Cr-1Mo-0.25V at room temperature, the half-life cycle is reproduced satisfactorily at strain ranges of Δε=1% and 0.80%, whereas larger discrepancies are observed at Δε=0.60% and 0.40%. This trend is observed in nearly all the tests performed on this material, with the exception of the cycles at 400 °C, for which the agreement is significantly better.

The reduced accuracy at lower strain ranges can be attributed to limitations inherent to the Chaboche model adopted in this work. In particular, the model assumes that the stress peaks corresponding to different strain ranges lie on a single master curve, in accordance with Masing behaviour. However, some materials, particularly stainless steels [1,26], exhibit non-Masing behaviour over certain strain ranges. Since the 1Cr-1Mo-0.25V investigated here is a martensitic stainless steel, such effects may contribute to the observed discrepancies. The inability of the model to accurately reproduce the stress peaks at lower strain ranges therefore indicates the limitations of the adopted formulation and the potential need for a more advanced constitutive model. This behaviour is particularly evident in Figure 4a and Figure 9a.

If the maximum and minimum strain levels expected during the service life of a component are known, the proposed fitting procedure can therefore be applied to the hysteresis cycle corresponding to the largest strain range expected in service. This ensures that the estimated parameters provide the most accurate representation of the material response within the relevant operating range.

More advanced constitutive models capable of capturing these features generally do not lend themselves to simple analytical relationships, preventing the development of similarly straightforward parameter-evaluation procedures and requiring more sophisticated numerical optimization or trial-and-error approaches. Moreover, such models are not always available in commercial FE software. This is particularly relevant for complex components with multiple notches subjected to thermo-mechanical loading, where even the Chaboche–Voce model can entail significant computational costs and potential convergence issues. Of course, the proposed approach can be regarded as a first step towards more advanced evaluation procedures and more sophisticated constitutive models.

The use of the Voce model for isotropic hardening proves effective in most of the cases investigated, with the exception of 1Cr-1Mo-0.25V at 500 °C. In this case, although the half-life cycle is reproduced reasonably well, a systematic discrepancy is observed in the plastic knee of the N=1 cycle. The adoption of an isotropic hardening model with a variable γ leads to improvements, but only in cases with a larger strain range.

Finally, it should be noted that the proposed methodology requires further validation through additional experimental testing. This should include stress-controlled tests at different mean stress levels, strain-controlled tests at different mean strain levels, and thermo-mechanical tests to assess the practical applicability of the estimated parameters when implemented in the computational framework.

## 5. Conclusions

The main conclusions of this study can be summarized as follows:

- An efficient parameter-evaluation procedure for the Chaboche model was proposed, based on an analytical relationship involving the asymptotic value of the backstress and requiring only a simple least-squares fit, with negligible computational cost.
- Isotropic hardening can be modelled either using the Voce formulation or by introducing a variable γ that depends on the accumulated plastic strain. In both cases, a rapid evaluation procedure based on the peak tensile stress in the first cycle allows the model parameters to be determined.
- The proposed procedure was applied to 1Cr-1Mo-0.25V steel at different temperatures and to 42CrMo4+QT steel at RT with two different values of total mean strain.
- The estimated parameter sets proved effective in reproducing the first cycle, the half-life cycle, and the evolution of the tensile and compressive peak stresses throughout the cyclic loading history.
- The investigated materials exhibit strain-range-dependent behaviour that prevents the basic Chaboche–Voce model from fully capturing certain aspects of the cyclic response. Nevertheless, the results demonstrate that the proposed procedure provides practically useful parameter sets for rapid implementation in FE analyses of complex structures, requiring only a simple least-squares fitting procedure.
- Future developments may include the evaluation of parameters for unified viscoplastic models. In this case, the Chaboche parameters may differ from those estimated using the present procedure, as the contribution of the viscous stress, $\sigma_{v}$, must also be accounted for in the yield function.
- Further improvements are required for the application to thermo-mechanical cycles, particularly to ensure a smooth and physically consistent temperature dependence of the $C_{i}$ parameters and, consequently, of their derivatives with respect to temperature.

## Figures and Tables

**Table 1:** Chemical composition (wt %) of 42CrMo4+QT steel from [39].

C	Si	Mn	P	S	Ni	Cr	Mo	V	Al	Ti	Sn	Fe
0.42	0.21	0.64	0.013	0.009	0.06	1.04	0.185	0.006	0.026	0.001	0.003	Balance

**Table 2:** Chemical composition requirements (wt %) of 1Cr-1Mo-0.25V steel.

C	Si	Mn	P	S	Ni	Cr	Mo	V	Fe
0.25–0.35	0.15–0.35	≤1.00	≤0.015	≤0.018	≤0.75	0.90–1.50	1.00–1.50	0.20–0.30	Balance

**Table 3:** Details regarding the testing campaign for both materials under investigation.

Description	Strain History Details
**Material**: 1Cr-1Mo-0.25V	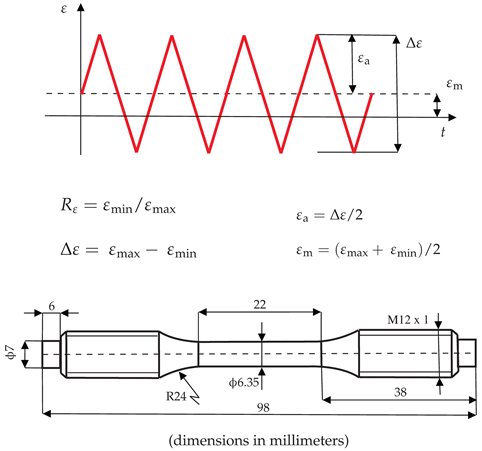
**T = 20 °C**	
Δε=0.40,0.60,0.80,1.00%	
$\dot{\varepsilon }=1\times 10^{-3}$/s, $R_{\varepsilon }=-1$	
**T = 200 °C**	
Δε=0.40,0.60,0.80,1.00%	
$\dot{\varepsilon }=1\times 10^{-3}$/s, $R_{\varepsilon }=-1$	
**T = 400 °C**	
Δε=0.40,0.60,0.80,1.00%	
$\dot{\varepsilon }=1\times 10^{-3}$/s, $R_{\varepsilon }=-1$	
**T = 500 °C** [27]	
Δε=0.40,0.60,1.00,1.30%	
$\dot{\varepsilon }=3\times 10^{-3}÷6\times 10^{-3}$/s, $R_{\varepsilon }=-1$	
**Material**: 42CrMo4+QT [38]	
**T = 20 °C**	
Δε=1.00,1.50,2.00,2.50%	
$\dot{\varepsilon }=1\times 10^{-2}$/s, $R_{\varepsilon }=-1$	
**T = 20 °C**	
Δε=1.00,1.50,2.00,2.50%	
$\dot{\varepsilon }=1\times 10^{-2}$/s, $\varepsilon_{m}=0.50\%$	

**Table 4:** Values of the Chaboche–Voce parameters and elastic constants for the material 42CrMo4+QT. For brevity, unless otherwise specified, in this paper 42CrMo4 refers to 42CrMo4+QT.

Material	$C_{1}$, GPa	$\gamma_{1}$	$C_{2}$, GPa	$\gamma_{2}$	$C_{3}$, GPa	$\gamma_{3}$	*k*, MPa	*Q*, MPa	*b*	*E*, GPa
42CrMo4	433	2462	75	322	18	1	331	−197.4	0.80	210

**Table 5:** Values of the Chaboche–Voce parameters and elastic constants for the materials studied at various temperatures.

Material	T = 20 °C	T = 200 °C	T = 400 °C	T = 500 °C
1Cr-1Mo-0.25V	$C_{1}=358$ GPa	$C_{1}=664$ GPa	$C_{1}=1810$ GPa	$C_{1}=88.2$ GPa
	$\gamma_{1}=4916$	$\gamma_{1}=9321$	$\gamma_{1}=$ 10,031	$\gamma_{1}=6734$
	$C_{2}=81.8$ GPa	$C_{2}=124$ GPa	$C_{2}=140$ GPa	$C_{2}=238$ GPa
	$\gamma_{2}=642$	$\gamma_{2}=1040$	$\gamma_{2}=1432$	$\gamma_{2}=639$
	$C_{3}=14.9$ GPa	$C_{3}=21.8$ GPa	$C_{3}=15.3$ GPa	$C_{3}=1.180$ GPa
	$\gamma_{3}=1$	$\gamma_{3}=1$	$\gamma_{3}=1$	$\gamma_{3}=1$
	k=226 MPa	k=140 MPa	$k=3.28\cdot 10^{-13}$ MPa	$k=8.10\cdot 10^{-12}$ MPa
	Q=−118.6 MPa	Q=−129 MPa	Q=−156 MPa	Q=−121 MPa
	b= 0.15	b=0.18	b=0.23	b=1.10
	E=210 GPa	E=200 GPa	E=186 GPa	E=146 GPa

## Data Availability

The original contributions presented in this study are included in the article. Further inquiries can be directed to the corresponding authors.

## Abbreviations

The following abbreviations are used in this manuscript:

ASTM	American Society for Testing and Materials
FE	Finite Element
GA	Genetic Algorithm
LCF	Low-Cycle Fatigue
MCMC	Markov Chain Monte Carlo
QT	Quenched and Tempered
RT	Room Temperature

## Nomenclature

The following nomenclature is used in this manuscript (Latin letters first, then Greek letters, in alphabetical order):

$A_{i}$	Asymptotic value of the *i*-th backstress, $A_{i}=C_{i}/\gamma_{i}$ (MPa)
*b*	Voce isotropic hardening rate parameter (−)
$C_{i}$	Kinematic hardening modulus of the *i*-th backstress (GPa)
*E*	Young’s modulus (GPa)
*f*	Yield function (MPa)
*k*	Saturated yield stress, $k=\sigma_{y,0}+Q$ (MPa)
*N*	Cycle number (−)
$N_{f}$	Number of cycles to failure (−)
*p*	Accumulated plastic strain (−)
$\dot{p}$	Accumulated plastic strain rate ($s^{-1}$)
*Q*	Voce isotropic hardening saturation parameter (MPa)
*R*	Isotropic hardening variable, Voce (MPa)
R^2^	Coefficient of determination (−)
$R_{\varepsilon }$	Strain ratio, $R_{\varepsilon }=\varepsilon_{\min}/\varepsilon_{\max}$ (−)
*T*	Temperature (°C)
$\dot{T}$	Temperature rate (°C $s^{-1}$)
$\gamma_{i}$	Dynamic recovery parameter of the *i*-th backstress (−)
$\gamma_{1,0}$	Value of $\gamma_{1}$ at the first cycle, p=0 (−)
Δε	Total strain range (−)
$\Delta \varepsilon_{\mathrm{pl}}$	Plastic strain range of a given stabilized cycle (−)
$\Delta \varepsilon_{\mathrm{pl},\mathrm{stab}}$	Plastic strain range of the stabilized cycle (−)
$\varepsilon_{m}$	Mean strain (−)
$\dot{\varepsilon }$	Total strain rate ($s^{-1}$)
$\varepsilon_{\mathrm{pl}}$	Plastic strain (−)
${\dot{\varepsilon }}_{\mathrm{pl}}$	Plastic strain rate ($s^{-1}$)
$\varepsilon_{\mathrm{pl},0}$	Initial plastic strain, integration constant (−)
$\varepsilon_{\mathrm{pl},1}^{\max}$	Maximum plastic strain reached in the first cycle (−)
σ	Applied stress (MPa)
$\sigma_{y,0}$	Initial yield stress (MPa)
$\sigma_{1}^{\max}$	Tensile peak stress of the first cycle (MPa)
$\sigma_{N}^{\max}$	Tensile peak stress of cycle *N* (MPa)
$\sigma_{\mathrm{stab}}^{\max}$	Tensile peak stress of the stabilized (half-life) cycle (MPa)
$\sigma_{v}$	Viscous stress (MPa)
$\Phi_{i}\left(p\right)$	Function scaling $\gamma_{i}$ (−)
$\Phi_{i,\infty }$	Saturated value of $\Phi_{i}$ (−)
$\chi_{\mathrm{tot}}$	Total backstress (MPa)
$\chi_{i}$	*i*-th backstress component (MPa)
$\chi_{i,0}$	Initial value of the *i*-th backstress (MPa)
$\chi_{i,\mathrm{stab}}^{\min}$, $\chi_{i,\mathrm{stab}}^{\max}$	Minimum/maximum stabilized *i*-th backstress (MPa)
ω	Evolution-rate parameter of $\Phi_{i}$ (−)
